# Cathepsin D Variants Associated With Neurodegenerative Diseases Show Dysregulated Functionality and Modified α-Synuclein Degradation Properties

**DOI:** 10.3389/fcell.2021.581805

**Published:** 2021-02-11

**Authors:** Josina Bunk, Susy Prieto Huarcaya, Alice Drobny, Jan Philipp Dobert, Lina Walther, Stefan Rose-John, Philipp Arnold, Friederike Zunke

**Affiliations:** ^1^Institute of Biochemistry, Christian-Albrechts-Universität zu Kiel, Kiel, Germany; ^2^Department of Molecular Neurology, University Hospital Erlangen, Friedrich-Alexander-Universität Erlangen-Nürnberg, Erlangen, Germany; ^3^Institute of Anatomy, Christian-Albrechts-Universität zu Kiel, Kiel, Germany; ^4^Institute of Anatomy, Functional and Clinical Anatomy, Friedrich-Alexander-University Erlangen-Nürnberg, Erlangen, Germany

**Keywords:** lysosomal degradation, molecular dynamics simulation, Parkinson’s disease, neuronal ceroid lipofuscinoses, lysosomes, alpha-synuclein, cathepsin D

## Abstract

Cathepsin D (CTSD) is a lysosomal protease important for the degradation of various substrates, including disease-associated proteins like α-synuclein (a-syn), amyloid precursor protein (APP) and tau, all of which tend to aggregate if not efficiently degraded. Hence, it is not surprising that genetic variants within the *CTSD* gene have been linked to neurodegenerative diseases, like Parkinson’s and Alzheimer’s disease (PD, AD), as well as the lysosomal storage disorder neuronal ceroid lipofuscinosis type-10 (NCL10). Although recent studies have shown the molecular dependence of substrate degradation via CTSD within autophagic pathways, only little is known about the precise role of lysosomal CTSD function in disease development. We here performed biochemical, cellular and structural analyses of eleven disease-causing CTSD point mutations found in genomic sequencing data of patients to understand their role in neurodegeneration. These CTSD variants were analyzed for cellular localization, maturation and enzymatic activity in overexpression analyses. Moreover, for PD-associated mutants, intracellular degradation of a-syn was monitored. In summary, our results suggest that NCL10-associated CTSD variants are significantly impaired in lysosomal maturation and enzymatic activity, whereas the AD- and PD-associated variants seemed rather unaffected, indicating normal maturation, and lysosomal presence. Interestingly, a PD-associated CTSD variant (A239V) exhibited increased enzymatic activity accompanied by enhanced a-syn degradation. By structural analyses of this mutant utilizing molecular dynamics simulation (MDS), we identified a structural change within a loop adjacent to the catalytic center leading to a higher flexibility and potentially accelerated substrate exchange rates. Our data sheds light onto the role of CTSD in disease development and helps to understand the structural regulation of enzymatic function, which could be utilized for targeted CTSD activation. Because of the degradative function of CTSD, this enzyme is especially interesting for therapeutic strategies tackling protein aggregates in neurodegenerative disorders.

## Introduction

The aspartic protease Cathepsin D (CTSD) is involved in the lysosomal recycling and degradation of many substrates, including the Parkinson’s disease (PD)-associated protein α-synuclein (a-syn) (Sevlever et al., 2008). CTSD is ubiquitously expressed, being particularly abundant in the brain (Stoka et al., 2016). Several genomic variants of *CTSD*, like coding missense mutations, seem to be related to neurodegenerative diseases, such as neuronal ceroid lipofuscinosis (NCL), Alzheimer’s disease (AD), and PD, all progressive disorders involving the central nervous system (CNS) and aggregation of misfolded proteins (Siintola et al., 2006; Steinfeld et al., 2006; Fritchie et al., 2009; Hersheson et al., 2014; Robak et al., 2017). Interestingly, the exact role of CTSD dysfunction in disease progression as well as its therapeutic potential in neurodegenerative disorders is not well understood.

Numerous studies highlight the functional role of CTSD in neurogenesis and neuronal communication (Partanen et al., 2008; Koch et al., 2011), as well as its pivotal role in lysosomal proteolysis, since CTSD dysfunction has been shown to lead to the accumulation of non-degraded substrates in the lysosomes (Cullen et al., 2009; Bae et al., 2015; Marques et al., 2019). As lysosomal dysfunction and substrate aggregation is especially toxic to postmitotic cells, like neurons, it is not surprising that many lysosomal storage disorders present with neurological impairments and vice versa, many neurodegenerative diseases are characterized by lysosomal dysfunction (Klein and Mazzulli, 2018; Wallings et al., 2019; Zunke, 2020). This underlines the importance of intact lysosomal enzyme function especially within neuronal cells.

Before CTSD can act as lysosomal protease, it undergoes a gradual maturation via the secretory pathway, being synthesized as an inactive precursor pro-enzyme comprising 412 amino acids (aa). After removal of the N-terminal signal peptide within the endoplasmic reticulum (ER), the inactive proCTSD (∼52 kDa) is glycosylated and transported to the Golgi compartment, where the mannose residues are phosphorylated for targeting to lysosomes via the mannose-6-phosphate pathway. An acidic environment is crucial for proteolytic processing and maturation of CTSD. Once in the endosomes, the phosphate groups and pro-peptide of 44 aa are removed to generate an active intermediate (∼48 kDa). In the lysosomal compartment, this single-chain intermediate is further processed into a double-chain mature form consisting of an N-terminal light chain (∼14 kDa) and a C-terminal heavy chain (∼34 kDa), which both remain non-covalently associated (Zaidi et al., 2008). This highly complex CTSD processing pathway depicts its vulnerability and potential effect of CTSD mutations on maturation and enzymatic function.

NCLs are a group of autosomal recessive neurodegenerative lysosomal storage disorders, and are the most common cause of pediatric neurodegenerative disease (Vidoni et al., 2016). The pathological features include the presence of autofluorescent storage bodies consisting of lipofuscin in lysosomes of neuronal and glial cells (Koike et al., 2000), resulting in a severe and progressive loss of motor and psychological abilities (Mink et al., 2013).

Congenital NCL type-10 (NCL10), the most severe form of the disease, is caused by mutations within the *CTSD* gene (Siintola et al., 2006). Moreover, increasing evidence suggests that CTSD is also implicated in processing of amyloid precursor protein (APP) (Zhou et al., 2006) and the tau protein (Ladror et al., 1994; Kenessey et al., 1997; Vidoni et al., 2016). Both proteins are linked to AD progression, which is characterized by the extracellular deposits of the amyloid β-protein (proteolytically processed APP) and intracellular neurofibrillary tangles of hyperphosphorylated tau protein (Selkoe, 2001).

Moreover, *in vitro* and *in vivo* studies demonstrated that under physiological conditions, CTSD mediates the lysosomal proteolysis of a-syn (Sevlever et al., 2008; Cullen et al., 2009; McGlinchey and Lee, 2015). It was shown that CTSD deficiency facilitates a-syn-dependent toxicity (Cullen et al., 2009). a-Syn aggregation is major hallmark of PD, the second most common neurodegenerative disorder (Kalia and Lang, 2015; Riederer et al., 2019). Recently, damaging variants of CTSD were found to be genetically linked to lysosomal dysfunction and PD pathology in a large screening of PD patients (Robak et al., 2017), further emphasizing the importance of its lysosomal clearance function (Klein and Mazzulli, 2018; Zunke, 2020).

In this study, we characterize eleven CTSD variants associated with neurodegenerative diseases by analyzing their cellular localization, maturation, enzymatic activity, a-syn degradation capacity and structural properties. Our findings contribute to a better understanding of CTSD regulation as well as its role in disease pathology and a-syn degradation pathways.

## Materials and Methods

### cDNA Constructs and Cloning

Expression plasmids of human (h) CTSD wildtype (wt), the disease-associated CTSD mutants (A58V, S100F, G149V, F229I, Y255X, W383C, R399H, V95I, G145V, A239V, and R266H) as well as the inactivecontrol D97S were cloned via site-directed mutagenesis PCR utilizing the hCTSD in the pCMV6/pcDNA3.1 vector as a template. All plasmidswere verified by DNA sequencing (GATC Biotech). A plasmid of hα-syn (pcDNA3.1) was used for co-transfection together with CTSD plasmids for further analyses of CTSD activity.

### Cell Culture

SH-SY5Y neuroblastoma wt and CTSD KO cells were maintained in advanced Dulbecco’s Modified Eagle’s medium/F12 (DMEM) (Thermo 611 Fisher Scientific; #12634010) containing 10% heat-inactivated fetal calf serum (FCS) and 1% penicillin/streptomycin (Pen/Strep) (PAA Laboratories GmbH; #P11-010). The human neuroglioma (H4) and the H4 CTSD KO cell line, both expressing a-syn under the control of a tetracycline inducible promoter (tet-off system), were cultured in OptiMEM media (Thermo Fisher Scientific; #31985070) containing 5% FCS, 200 μg/ml Geneticin (Thermo Fisher Scientific; #10131035) Hygromycin (Sigma-Aldrich, #H7772-1g), and 1% Pen/Strep.

### iPS Cells Cultivation (2135) and Differentiation Into Dopaminergic Neurons

Human induced pluripotent stem (iPS) cell line (2135) from a healthy control donor was established and characterized previously (Seibler et al., 2011). iPS cells were maintained on matrigel (Corning; #354234)-coated dishes with mTeSR1 media (Stemcell Technologies; #85850) and passaged once per week. iPS cells were differentiated into midbrain dopaminergic (DA) neurons (iPSn) by using a mixture of growth factors as described previously (Kriks et al., 2011). In brief, iPS cell colonies were enzymatically dissociated and seeded onto matrigel-coated 12 well dishes. When cells reach a confluency of 80%, the differentiation protocol was initiated by adding knockout serum replacement (KSR) media with dual SMAD inhibitors and carried out for 15 days with the addition of growth and differentiation factors as depicted in detail in Kriks et al. (2011). Between day 10 and 15, cell layers were mechanically dissociated into small squares of ca. 2 mm^2^ and plated onto a 6-well dish, coated with poly-d-lysine (PDL, 33 μg/mL) and 5 μg/mL laminin. After 25–30 days, the cells were detached by accutase (Corning; #25-085-Cl), counted, and plated at a cell number of 4 × 10^5^ cells for western blot analysis on PDL/laminin-coated 24-well plates. The growth factors were withdrawn at day 40–50 and cells were aged until day > 80. iPS-derived DA neurons were maintained in neurobasal media (Thermo 611 Fisher Scientific, #21103-049) containing NeuroCult SM1 supplement (Stemcell Technologies, 612 #05711), 1% L-Glutamine (200 mM stock, Gibco; #25030-081), and 1% Pen/Strep (Sigma-Aldrich; 613 #P0781). The quality of DA neurons was regularly validated by the presence of neuronal and midbrain dopamine markers (FOXA2, β-iii-tubulin, tyrosine hydroxylase and synapsin) by immunostaining and western blot.

### Generation of CTSD Knockout (KO) in SH-SY5Y and H4 Cells by CRISPR/Cas9

SH-SY5Y neuroblastoma cells and H4 neuroglioma cells were seeded 2 days before transfection, so that 70–80% confluency was reached on the day of transfection. Cells were washed with PBS and were dissociated from the plate by trypsinization (0.5% Trypsin-EDTA, Gibco, #25300-054). The reaction was neutralized by adding the 2X volume of normal growth medium. Cell density was determined by using a cell counter (Nexcelom Bioscience; #SD100) and cells were transferred to a sterile 1.5 mL microfuge tube after counting. Each transfection required 50,000 cells. Cells were centrifuged at 500 × *g* for 5 minutes and washed again with PBS. After repeated centrifugation, cells were resuspended in 5 μL resuspension buffer R (Neon Transfection System 10 μL-Kit; Invitrogen; #MPK1025) per transfection. RNP (ribonucleoprotein) complexes of CTSD multi RNA guides and Cas9 protein were assembled as given in the manufacturer’s protocol (Gene Knockout Kit v2; Synthego; United States). The multi RNA guides used in this study target the exon 2 of the *CTSD* gene. Guide RNA (gRNA) 1: 5′-UAGUUCUUGAGCACCUC-3′, gRNA 2: 5′-CUCAAAGUACUCCCAGG-3′, and gRNA 3: 5′-ACCAUGUCGGAGGUUGG-3′. 5 μL of cell suspension was added to each RNP solution. 10 μL of total cell-RNP solution was filled into a 10 μL Neon tip (Neon Transfection System 10 μL-Kit; Invitrogen; #MPK1025) and transfected via electroporation (Neon Transfection System; Invitrogen) at 1200 V; 20 ms and 3 pulses. Cells were transferred immediately to a pre-warmed 6-well dish contanining growth media without antibiotics and incubated until analysis of mixed pooled CTSD KO cells. Successful editing efficiency was determined by western blot analysis and Sanger sequencing of *CTSD* exon 2. For Sanger sequencing analysis, genomic DNA was isolated by DNeasy Blood and Tissue Kit from Qiagen (Qiagen; #69504). Following primers were used to amplify *CTSD* exon 2 of pooled CTSD KO cells and wt cells: forward 5′-GCAGGAGTTTGGTTTTGGCT-3′ and reverse 5′-ACTCCCAATCACCCTCCCAG-3′. Genomic DNA was amplified using Q5 High-Fidelity DNA polymerase (New England Biolabs; # M0491L) by the following PCR protocol: 98°C for 30 s, 35 cycles of (98°C for 10 s, 65°C for 40 s, 72°C for 30 s), and 72°C for 5 min. The PCR product was analyzed by agarose gel electrophoresis using ethidium bromide-stained 1.5% agarose gel. The PCR product of the correct size was excised from the gel and DNA was extracted by using a GeneJET Gel-Extraction Kit (Thermo Scientific; #K0692). For Sanger sequencing, the following primers were used: 5′- GCAGGAGTTTGGTTTTGGCT-3′. In order to get single cells, CTSD KO cells were diluted and seeded at one cell per 96-well plate. Single CTSD KO cells were grown and expanded for western blot and Sanger sequencing analysis. The same procedure was performed to check successful genome editing in single cell clones.

### Transfection and Inhibitor Treatment

In order to ensure reproducibility, the cell count was determined prior to each transfection by Cellometer^®^ Auto T4 Plus (Nexcelom Bioscience; #SD100). SH-SY5Y CTSD KO cells were seeded into 6-well plates at 2 × 10^5^ cells per well for western blot analysis and in same density onto 12 mm cover glasses for immunofluorescence analyses. After 24 h of expression, cells were transiently transfected with Lipofectamine 2000 (Thermo 611 Fisher Scientific; #11668027) following the manufacturer’s protocol. Cells were washed in PBS and harvested for experimental analysis after 48 h and a-syn co-transfected cells were harvested after 72 h of expression. H4 CTSD KO cells were plated at a density of 4 × 10^5^ per well (6-well plate) and transfected 24 h later using Effectene transfection reagent (Qiagen; #301425) according to the manufacturer’s instructions. H4 CTSD KO cells were harvested after 48 h of expression. For CTSD inhibition, pepstatin A (PepA; Sigma-Aldrich; #508437) was diluted in cell culture media to a final concentration of 100 μM and incubated for 2 days after transfection. Bafilomycin A1 (BafA1) was used to inhibit the lysosome. BafA1 (Santa Cruz; #sc-201550A) was dissolved in DMSO and diluted in cell culture media to a final concentration of 0.2 μM. Cells were treated with BafA1 one day after transfection for 16 h.

### Immunofluorescence Analysis

SH-SY5Y CTSD KO cells were fixed in 4% paraformaldehyde (PFA) for 15 min at 37°C, permeabilized with 0.3% Triton X-100 (Roth; #3051.2) in PBS-Triton for 30 min, then blocked in 2% BSA, 5% heat-inactivated FCS in PBS-Triton (blocking buffer) for 1 h. The primary antibodies were diluted in blocking buffer to their working concentration (see below) and incubated at 4°C overnight. Cells were washed three times with PBS-Triton and incubated with secondary antibodies diluted 1:500 in blocking buffer. Afterwards, cells were washed three times with PBS-Triton, once with PBS and stained with DAPI Fluoromount-G (SouthernBiotech; #SBA-0100-20). Immunofluorescence analyses were performed with a confocal laser scanning microscope (IX83, Olympus) equipped with a U Plan S Apo 100X oil immersion objective. Digital images were processed and analyzed using Inspector Image Acquisition and Analysis Software (Abberior Instruments). The Pearson’s correlation coefficient was used to express co-localization of two stainings. Transfected SH-SY5Y CTSD KO cells were marked by a region of interest and co-localization of two stainings was determined with the ImageJ software, resulting in values ranging from −1 to +1. Positive values describe a positive correlation between both signals (co-localization), negative values describe a negative correlation and a value of 0 indicates random distribution of both signals (Pearson, 1909). Quantification of LAMP2 was done semiautomatically by thresholding to a similar intensity for count and length measurement of LAMP2-positive vesicles in mock and transfected cells.

Primary antibodies used: anti-CTSD (1:100 BD Biosciences; #610801), anti-LAMP2 (1:250, DSHB, #H4B4). Secondary antibodies: goat-anti-mouse Alexa Fluor 594 (1:500, Thermo Fisher Scientific; #A11032) and goat-anti-rabbit Alexa Fluor 488 (1:500, Thermo Fisher Scientific; #A11037).

### Western Blot Analysis

All cells were mechanically detached and washed with ice-cold PBS. SH-SY5Y CTSD KO cell and H4 CTSD KO cell pellets were extracted in a Triton-based buffer (1% Triton X-100, 20 mM HEPES pH 7.4, 150 mM NaCl, 10% glycerol, 1 mM EDTA, 1.5 mM MgCl_2_) supplemented with 1 mM phenylmethanesulfonyl fluoride (PMSF), 50 mM sodium fluoride (NaF), 2 mM sodium orthovanadate (NaVO_3_), and a protease inhibitor cocktail (Roche) by incubation in an ice-water slurry for 20 min, followed by two freeze and thaw cycles, and ultracentrifugation at 100,000 × *g*, 4°C for 30 min. The soluble supernatant was then further analyzed. The amount of total protein was determined by bicinchoninic acid assay (BCA) (Thermo Fisher; #23227). Samples were denatured with 5 X Laemmli buffer (0.3 M Tris–HCl, pH 6.8, 10% SDS, 50% glycerol, 5% β-mercaptoethanol, 5% bromophenol blue) and heat inactivated at 95°C for 10 min. Equal amounts of protein (40 μg) were separated by electrophoresis on a 12% SDS-PAGE gel and transferred to PVDF-membranes (Merck Millipore; #IPFL00010). Membranes were blocked in a 1:1 mixture of TBS and blocking buffer (LI-COR Bioscience; #927-50003) for 1 h. For a-syn analysis, membranes were fixed in 0.4% PFA for 20 min before blocking. Primary antibodies were incubated overnight at 4°C. Detection was carried out with fluorescence-conjugated secondary antibodies (LI-COR Biosciences) and detected by an imaging system (Amersham Typhoon, GE Life Sciences).

Following antibodies were used for detection: anti-CTSD (1:500; BD Biosciences; #610801), anti-α-syn (Synuclein-1; 1:1000; BD Biosciences; #610787), and anti-GAPDH (1:1,000, Cell Signaling; #2118).

### Cell Lysate CTSD Activity Assay

SH-SY5Y CTSD KO and H4 CTSD KO cells overexpressing NCL10/AD- and PD-associated CTSD variants were lysed in a Triton-based buffer (50 mM sodium acetate, 0.1 M NaCl, 1 mM EDTA, 0.2% Triton X-100, pH 4.5) by shaking for 1 h at 4°C. Lysates were obtained by centrifugation and immediately used for determination of activity. 2 μl of cell lysates were incubated in lysis buffer containing 0.1 μM quenched fluorogenic peptide (Enzo; #BML-P145) and 0.05 mM Leupeptin (Enzo; #ALX-260-009-M025) at 37°C for 30 min. Recombinant CTSD [produced in-house, as recently described (Marques et al., 2019)] and samples treated with CTSD inhibitor pepstatin A (Sigma-Aldrich; #P5318-5MG) were used as positive and negative assay controls, respectively. CTSD activity was measured with an Infinite^®^ 200 PRO M Plex multimode microplate reader (ex.: 360 nm; em.: 440 nm; Tecan Trading AG; #TEC006418I). For every CTSD activity assay, expression of CTSD variants was verified by western blot. CTSD activity values were normalized to protein expression level and expressed relative to CTSD wt. Also, a catalytically inactive CTSD variant was established as control, replacing one of the aspartic acids (D) comprising the active site of the enzyme with a serine (S; D97S). This exchange of a carboxyl group with a hydroxyl group results in an enzymatically inactive CTSD that still showed normal maturation and lysosomal localization.

### Cell Lysate Cathepsin B (CTSB) Activity Assay

Cell lysates were produced as described above. 2 μl of the cell lysates were incubated with 20 μM fluorogenic CTSB substrate (Z-RR-AMC; Enzo; #BML-P137-0010) diluted in lysis buffer (50 mM sodium acetate, 0.1 M NaCl, 1 mM EDTA, 0.2% Triton X-100, pH 4.5) at 37°C for 30 min. Recombinant CTSB [produced in-house, as recently described for CTSD (Marques et al., 2019)] and the addition of the CTSB inhibitor Leupeptin (Enzo; #ALX-260-009-M025), were used as positive and negative controls. Detection was performed with an Infinite 200 PRO M Plex multimode microplate reader (ex.: 360 nm; em.: 440 nm; Tecan Trading AG; #TEC006418I).

### Cell Lysate β-Glucocerebrosidase (GCase) Activity Assay

Cell lysate extractions were carried out as described above. Next, 2 μl of the cell lysates were incubated with 1 mM fluorogenic substrate 4-methylumbellifery-β-D-glucopyranoside (4MU; Sigma-Aldrich; #M3633) diluted in 4MU buffer (150 mM citrate/phosphate, 0.25% sodium taurocholate, 0.25% Triton X-100, pH 5.4) at 37°C for 1 h. After incubation, equal amounts of stop solution (0.4 M Glycerol, pH 10,4) were added to the wells. Fluorescence signal was measured with an Infinite^®^ 200 PRO M Plex multimode microplate reader (ex.: 365 nm; em.: 445 nm; Tecan Trading AG; #TEC006418I).

### Lysosomal Staining

H4 CTSD KO cells (4 × 10^5^ cells) were seeded onto a 6-well and transfected on the following day (see protocol above). After 24 h of expression, cells were trypsinized and seeded onto a 96-well plate (3 × 10^4^ cells per 96 well). The next day, the cells were treated with 1 mg/ml Dextran, Cascade Blue^TM^ (Thermo Fisher Scientific; #D1976). The day after, cells were washed with PBS and the media was replaced by OptiMEM media without phenol red (Thermo Fisher Scientific; #11520386). Dextran blue signal, which indicates the lysosomal volume, was quantified at an excitation wavelength of 400 nm and emission wavelength of 430 nm. In order to get an accurate normalization of the Dextran blue signal, normalization to the cell marker celltag700 (LI-COR; # 926-41090) was performed. For this, cells were washed in PBS and fixed in 4% PFA for 20 min at RT. Afterwards, cells were washed again in PBS, permeabilized in 0.3% Trition X-100 in PBS and blocked in blocking buffer (see section “Immunofluorescence Analysis”). Next, celltag700 was added at a dilution of 1:500 in blocking buffer for 1 h at RT. The plate was then washed three times in 0.1% Tween20 in PBS. The last wash was conducted in PBS and celltag700 signal was detected by an imaging system (Amersham Typhoon; GE Life Sciences).

### Structural Analysis

As a template for structural analysis, the available structure of a CTSD dimer with inhibitor 2-(3,4-dimethoxyphenyl)-N-[N-(4-methylbenzyl)carbamimidoyl]acetamide was used (PDB: 4OBZ). For the model, only one CTSD molecule was used and the inhibitor was removed. Moreover, the light chain was colored light blue and the heavy chain was colored gray. The active site was highlighted red. Mutated amino acids were marked in different colors.

Molecular dynamics simulation (MDS) was performed as described before (Schneppenheim et al., 2017). In short, after removal of the inhibitor from the CTSD wt structure (PDB: 4OBZ), UCSF Chimera (Pettersen et al., 2004) was used to exchange alanine 239 to a valine (*swapaa*) and produce the A239V-CTSD. VMD (Humphrey et al., 1996) was used to prepare the structure for MDS in NAMD (Phillips et al., 2005). This preparation in VMD included the addition of a water box and the addition of NaCl-ions to neutralize the system. After 100,000 steps of minimization, 1,000,000 steps of simulation were calculated in NAMD. For CTSD wt and CTSD A239V, three independent approaches were calculated to assess structural differences between wt- and A239V-CTSD. All imaging was performed in UCSF Chimera.

### Data Analysis and Statistics

All values are expressed as mean ± SEM. For data analyses, Excel (Microsoft, Seattle, WA, United States) and GraphPad Prism version 7 (GraphPad Software for Mac, San Diego, CA, United States) were used. Differences among mean values were analyzed by one-way ANOVA, followed by a Tukey’s multiple comparison test. In all analyses, the null hypothesis was rejected at *p* < 0.05 with ^∗^ <0.05, ^∗∗^ <0.01, ^∗∗∗^ <0.001, ^****^ <0.0001.

## Results

To gain a better understanding of the role of CTSD in disease development, eleven *CTSD* point mutations found by genome sequencing analyses of NCL10, AD or PD patients were analyzed in overexpression studies by structural and functional readouts. In order to examine CTSD variants in neural-like cells, a CTSD knockout (KO) was established in human neuroblastoma (SH-SY5Y) and neuroglioma (H4) cell lines by CRISPR/Cas9 technology (Supplementary Figures 1A,B). Both cell lines are frequently used to study neurodegenerative disease pathways. Interestingly, SH-SY5Y and H4 cells exhibit much higher levels of endogenous CTSD in comparison to human embryonic kidney cells (HEK293T), but similar CTSD level as found within human dopaminergic (DA) neurons derived from induced pluripotent stem cells (iPSn) (Supplementary Figure 1B). CTSD protein level and cellular localization was similar and not significantly altered after overexpression in CTSD-deficient SH-SY5Y cells (CTSD KO) in comparison to endogenous CTSD within SH-SY5Y wt cells (Supplementary Figure 1C).

### NCL-Associated CTSD Variants Show Impaired Protein Maturation and Enzymatic Activity

**FIGURE 1:**
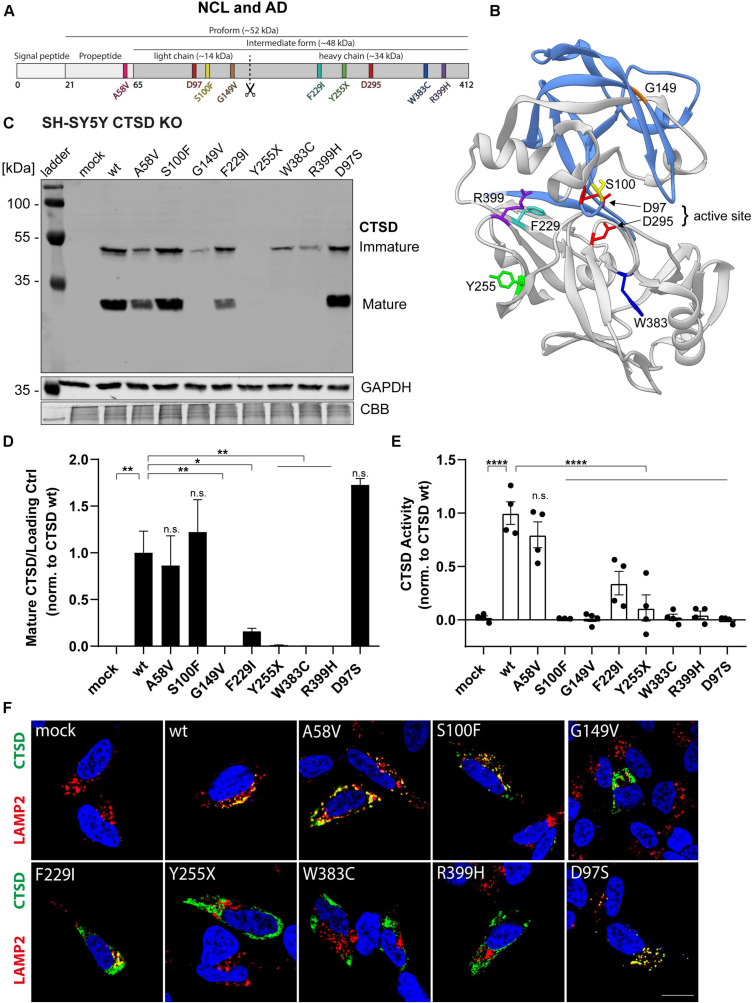
Characterization of CTSD variants associated with neurodegenerative diseases in SH-SY5Y CTSD KO cells. **(A)** Schematic overview of NCL- and AD-associated CTSD variants analyzed in this study. During protein maturation, the signal peptide (20 aa) and the propeptide (44 aa) are removed, generating an intermediate form (∼48 kDa) that is further processed in the lysosome into a double-chain mature form comprised of a light chain (∼14 kDa) and a heavy chain (∼34 kDa; symbolized by scissor symbol). Both chains remain associated by hydrophobic interactions. Point mutations found in NCL- and AD- patients located within different protein parts are shown in different colors (A58V, pink, propeptide; S100F, yellow, light chain; G149V, orange, light chain; F229I, light blue, heavy chain; Y255X, green, heavy chain; W383C, dark blue, heavy chain; R399H, purple, heavy chain). Aspartates D97 and D295 as part the catalytic site are highlighted in red. **(B)** Crystal structure model of mature CTSD consisting of the light chain (blue) and the heavy chain (gray) [PDB-ID: 4OBZ (Gradler et al., 2014)]. The active site, consisting of the two aspartates D97 and D295, is shown in red. Other colors indicate disease-associated point mutations within the CTSD protein (same color code as in **A**). **(C)** Representative immunoblot of transiently overexpressed CTSD wildtype (wt) and NCL-/AD-associated CTSD variants, as well as enzymatically inactive control (D97S) in SH-SY5Y CTSD KO cells. An anti-CTSD antibody was used for the detection of immature (pro- and intermediate form) as well as mature CTSD (heavy chain, 34 kDa). GAPDH and coomassie brilliant blue (CBB) were used as a loading control. **(D)** Quantification of western blot signal intensity of mature CTSD (heavy chain) normalized to GAPDH and expressed relative to CTSD wt (*n* = 4). **(E)** Analysis of CTSD activity assessed in whole cell lysates utilizing a fluorogenic CTSD peptide cleavage assay. The activity was normalized to CTSD wt (*n* = 4). **(F)** Representative immunofluorescence pictures of SH-SY5Y CTSD KO cells expressing CTSD wt or NCL-/AD-associated variants. Cells were visualized by staining of CTSD (green), the lysosomal associated membrane protein LAMP2 (red), and DAPI as nuclear staining (blue). Scale bar: 20 mm. Confocal images showing the single channels can be found in Supplementary Figure 2. All statistical analyses were performed using a one-way ANOVA followed by a Tukey’s multiple comparison test. *p < 0.05, **p < 0.01, ****p < 0.0001, n.s., not significant in comparison to wt.

Mutations within the *CTSD* gene have been linked to the severe neuropathic lysosomal storage disorder NCL10 (Siintola et al., 2006; Steinfeld et al., 2006; Fritchie et al., 2009; Doccini et al., 2016). For instance, a complete loss of CTSD function causes an early death of newborns, emphasizing the vital role of CTSD function (Siintola et al., 2006). A point mutation within the *CTSD* gene was also linked to the neurodegenerative disorder AD (Riemenschneider et al., 2006; Ehling et al., 2013). Interestingly, the course and severity of disease varies between the different genetic variants. To learn more about the effect of each CTSD point mutation, we characterized six NCL10- and one AD-linked CTSD mutation under the same experimental conditions by overexpression analyses (Figures 1A,B). The AD-associated CTSD mutant A58V is the only mutant found in the pro-peptide of CTSD (Figure 1A, pink), which gets cleaved during maturation and is not found within the mature protein. NCL-linked CTSD mutations are found within the light chain [S100F (yellow), G149V (orange)] and heavy chain [F229I (cyan), Y255X (green), W383 (blue), R399H (purple)] of the mature enzyme (Figures 1A,B). The structural representation of CTSD illustrates the localization of the S100F mutation in close proximity to the active site amino acid D97 (Figure 1B). In general, amino acid orientation of all here analyzed CTSD mutations are facing towards the inside of the protein, which might explain destabilizing effects on the protein (Figure 1B). For further analyses, a catalytically inactive CTSD mutant (D97S) was included as a control.

NCL10- and AD-associated CTSD variants were overexpressed in SH-SY5Y cells deficient for CTSD (CTSD KO) and immunoblot analyses were performed to analyze protein expression, stability as well as CTSD maturation: pro- (∼52 kDa) and intermediate form (∼48 kDa) were analyzed together as immature CTSD (Figure 1C). CTSD wt-transfected cells expressed the mature form of CTSD as depicted by the presence of the heavy chain (∼34 kDa) (Figure 1C). Only the AD-variant A58V, S100F and the inactive CTSD control (D97S) showed similar levels of the heavy chain compared to CTSD wt (Figures 1C,D). All other NCL-associated CTSD variants exhibited a significant reduction (F229I) or complete absence of mature CTSD (G149V, Y255X, W383C, R399H), indicating impaired protein maturation (Figures 1C,D). Next, the effects of the NCL- and AD-associated point mutations on enzymatic CTSD activity were assessed by a fluorogenic peptide cleavage assay of cell lysates (Figure 1E). All NCL-associated CTSD variants showed a significantly reduced enzymatic activity in comparison to CTSD wt, similar to the inactive CTSD control D97S (Figure 1E). In contrast, CSTD A58V exhibited similar enzymatic activity as the CTSD wt (Figure 1E).

Since lysosomal localization is crucial for CTSD maturation as well as for enzyme function, immunofluorescence studies were performed to analyze cellular localization of CTSD mutants. SH-SY5Y CTSD KO cells were transfected with respective CTSD variants and co-stained for CTSD (green) and lysosomal-associated membrane protein type 2 (LAMP2; red) as lysosomal marker (Figure 1F and Supplementary Figure 2). Co-localization and hence lysosomal localization of the CTSD variants was indicated by overlapping signals (yellow) (Figure 1F and Supplementary Figure 2). Calculating the co-localization of CTSD and LAMP2 via the Pearson’s correlation coefficient revealed no changes in lysosomal localization for CTSD A58V, S100F, and inactive D97S control (Supplementary Figure 3A). Confirming the immunoblot (Figures 1C,D), significantly reduced lysosomal localization was found for all NCL10-associated CTSD variants with the exception of CTSD S100F, indicating unimpaired maturation, and lysosomal localization (Figure 1F). Still, there was no enzymatic activity measured for the CTSD variant S100F (Figure 1E). Interestingly, the number of LAMP-2 positive vesicle was increased for the S100F mutant in comparison to the CTSD wt, whereas all other CTSD variants did not show any change in vesicle number (Supplementary Figure 3B). The average size of LAMP2 positive vesicles was increased for all CTSD variants located within the heavy chain of the CTSD protein (F291I, Y255X, W383C, R399H) in comparison to the wt (Supplementary Figure 3C).

Taken together, our results suggest that the AD-associated variant A58V follows a regular CTSD maturation and is enzymatically active, whereas all NCL10-associated variants exhibited impairments of either CTSD maturation and/or enzymatic function. Moreover, increased size of LAMP2-positive vesicles for the majority of NCL-associated CTSD variants indicates severe effects on lysosomal function.

### PD-Associated CTSD Variants Follow Unimpaired Protein Maturation and Enzymatic Activity

**FIGURE 2:**
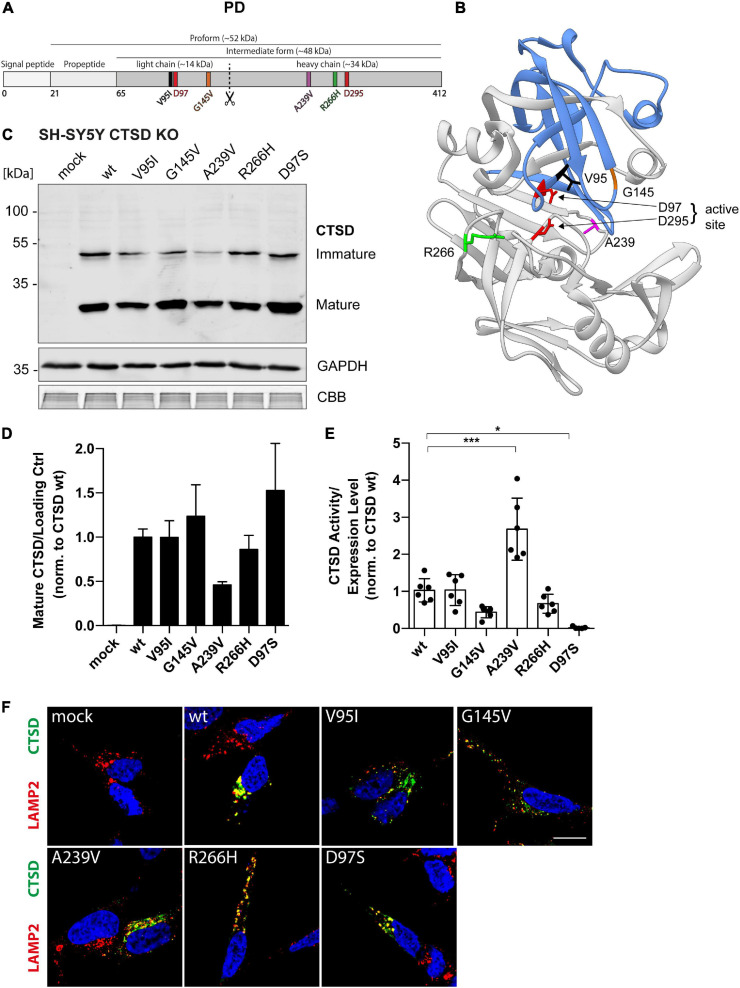
Structural and functional analyses of PD-associated CTSD variants in SH-SY5Y CTSD KO cells. **(A)** Schematic overview of the CTSD protein (412 aa) and its maturation intermediates (pro- and intermediate form, together referred to as immature form). Mature CTSD consists of a light chain (∼14 kDa) harboring two of the total four analyzed PD-associated mutations (V95I and G145V) and the heavy chain containing the A239V and R266H mutation. The aspartates D97 and D295 of the active site are shown in red. **(B)** Crystal structure model of mature CTSD protein consisting of the light chain (blue) and the heavy chain (gray) [PDB-ID: 4OBZ (Gradler et al., 2014)]. Active site residues (D97, D295) are shown in red. Other colors indicate PD associated point mutations (V95, black, light chain; G145, orange, light chain; R266, green, heavy chain; A239, purple, heavy chain). **(C)** Representative immunoblot of transiently overexpressed CTSD wildtype (wt), enzymatically inactive control (D97S), and PD-associated CTSD variants in SH-SY5Y CTSD KO cells. An anti-CTSD antibody was used for the detection of immature (pro- and intermediate form) as well as mature CTSD (heavy chain, 34 kDa). GAPDH and CBB were used as loading control. **(D)** Quantification of mature CTSD western blot signal intensity of CTSD variants normalized to loading control GAPDH and expressed relative to CTSD wt (*n* = 4). **(E)** CTSD activity was assessed in whole cell lysates by fluorogenic peptide cleavage assays and normalized to CTSD wt (*n* = 7). **(F)** Immunostaining analysis of CTSD (green) and lysosomal protein LAMP2 (red) in SH-SY5Y CTSD KO cells overexpressing CTSD wt or PD-associated variants. Nuclei are stained with DAPI (blue). Scale bar: 20 μm. More confocal images also showing single channels can be found in Supplementary Figure 4. All statistical analyses were performed by using a one-way ANOVA followed by a Tukey’s multiple comparison test. *p < 0.05, ***p < 0.001; n.s., not significant.

A recent whole exome sequencing analysis in 1156 PD cases and 1670 control subjects discovered four coding point mutations within the *CTSD* gene in PD patients (Robak et al., 2017). We here examined cellular and biochemical characteristics of those CTSD variants, found in the light chain (V95I, G145V) and heavy chain (A239V, R266H) of the mature enzyme (Figures 2A,B).

**FIGURE 3:**
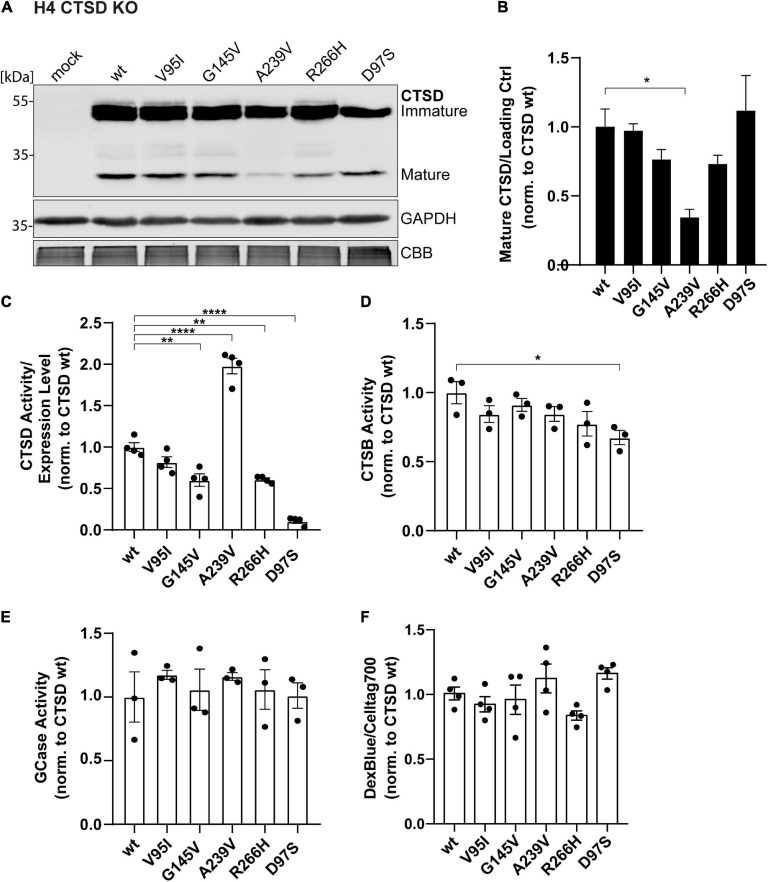
Cellular and functional analyses of PD-associated CTSD variants in H4 CTSD KO cells. **(A)** Representative immunoblot of CTSD wt, PD-associated CTSD variants (V95I, G145V, A239V, and R266H) and catalytically inactive mutant D97S overexpressed in neural-like H4 cells deficient for CTSD (CTSD KO). GAPDH and CBB were used as a loading control. **(B)** Analysis of CTSD protein expression by quantification of signal intensities of mature CTSD protein bands normalized to loading control (GAPDH) and expressed relative to CTSD wt (*n* = 4). **(C)** CTSD activity assay measured within H4 CTSD KO cell extracts and normalized to CTSD wt (*n* = 4). **(D)** Cathepsin B activity measured in whole cell lysates by fluorogenic peptide cleavage assays and normalized to CTSD wt (*n* = 3). **(E)** β-Glucocerebrosidase (GCase) activity measured in whole cell lysates and normalized to CTSD wt (*n* = 3). **(F)** Lysosomal homeostasis was assessed by measuring lysosomal mass using dextran blue (DexBlue) in a plate reader approach normalized to the cell marker celltag700. The values are shown as ratio to CTSD wt (*n* = 4). All statistical analyses were performed by using a one-way ANOVA followed by a Tukey’s multiple comparison test. *p < 0.05, **p < 0.01, ****p < 0.0001.

As CTSD is highly expressed in neuronal cell lines (Supplementary Figure 1B), where it degrades disease-associated substrates like a-syn and APP (Zhou et al., 2006; Sevlever et al., 2008; Cullen et al., 2009), we analyzed PD-associated CTSD variants in SH-SY5Y (Figure 2) as well as H4 cells (Figure 3) deficient for CTSD (CTSD KO). In SH-SY5Y CTSD KO, no significant difference in protein maturation was observed for the PD-associated CTSD variants in western blot analysis in comparison to wt, although mature protein level were less for the A239V CTSD variant (Figures 2C,D). Next, enzymatic activity of PD-associated CTSD variants was examined and no functional impairment could be detected (Figure 2E). Surprisingly, the CTSD A239V variant showed a 2.5-fold increased activity compared to CTSD wt (Figure 2E). In line with the western blot analyses (Figure 2C), immunofluorescence studies showed lysosomal localization of all PD-associated CTSD variants as indicated by co-localization with lysosomal marker LAMP2 (Figure 2F and Supplementary Figure 4), also confirmed by Pearson’s correlation analysis assessing overlay of LAMP2 and CTSD signal (Supplementary Figure 5A). Moreover, there was no change in number or average size of LAMP2-positive vesicles between wt and CTSD variants (Supplementary Figures 5B,C).

Overexpression of CTSD PD-mutants in H4 CTSD KO cells exhibited similar expression and maturation patterns as observed in SH-SY5Y CSTD KO cells (Figure 3A). However, in this cell line, the A239V variant showed a significant reduction of mature protein in comparison to the CTSD wt (Figures 3A,B). Yet, mature CTSD protein normalized to immature CTSD level did not exhibit any differences within PD-associated CTSD variants in both cell lines (SH-SY5Y CTSD KO and H4 CTSD KO; Supplementary Figures 5D,E). This suggests unimpaired maturation of all PD-CTSD variants. In line with the results in SH-SY5Y CTSD KO cells, a significant increase (2-fold) in enzyme activity was also measured in H4 CTSD KO cells transfected with the A239V mutant (Figure 3C).

To analyze possible cellular effects of CTSD variants on general cell and lysosomal homeostasis, activity of two other lysosomal enzymes, comprising cathepsin B (CTSB), and β-glucocerebrosidase (GCase) were measured in cell lysates. For CTSB, only the inactive CTSD D97S variant showed a significant reduction in CTSB activity in comparison to the wt (Figure 3D). Also, the GCase activity assay revealed no differences for PD-associated CTSD variants (Figure 3E). Lysosomal mass as marker for lysosomal dysfunction was determined by Dextran blue staining in a live cell plate reader assay, exhibiting no differences between the different CTSD variants (Figure 3F).

Collectively, these findings demonstrate that protein maturation, lysosomal localization, as well as enzymatic activity are not significantly impaired in PD-associated CTSD variants in SH-SY5Y CTSD KO and H4 CTSD KO cells. Moreover, no impairment in other lysosomal enzyme function and lysosomal homeostasis was observed in the H4 CTSD KO cell system after overexpression of any CTSD variant (wt, PD-associated or inactive control). Remarkably, CTSD variant A239V leads to an enhanced activity, suggesting a structural change within the protein, which will be analyzed in more detail in the following.

### PD-Associated CTSD A239V Shows Enhanced α-Synuclein Degradation

**FIGURE 4:**
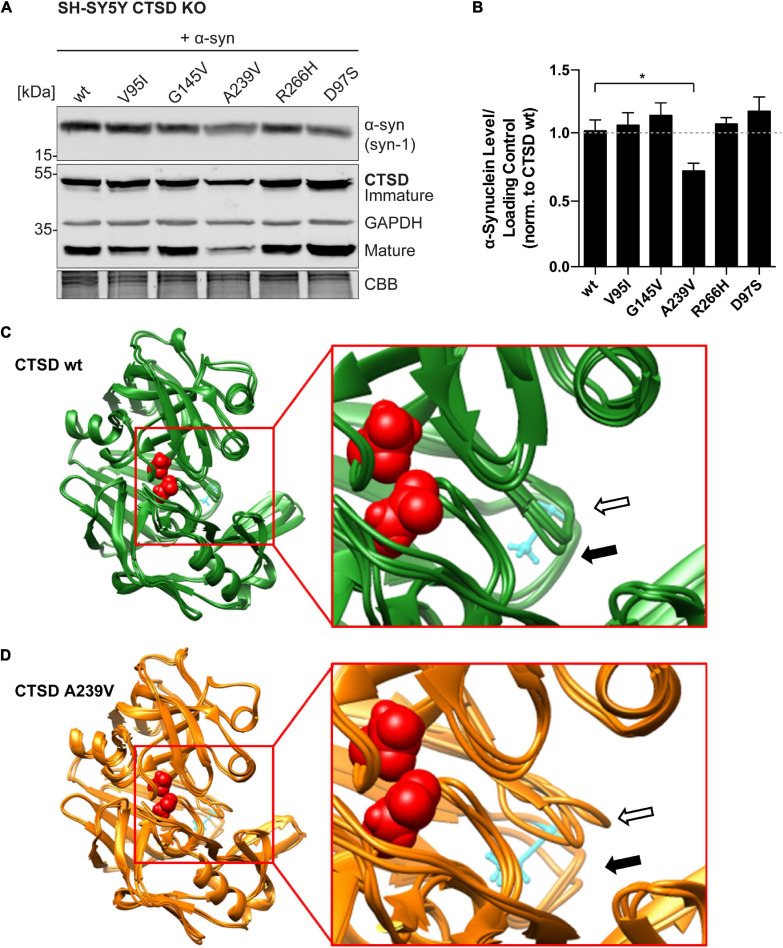
Functional and structural impact of PD-associated CTSD A239V mutation. **(A)** Immunoblot of CTSD variants co-expressed with a-syn in SH-SY5Y CTSD KO cells. Synuclein-1 (Syn-1) was used for a-syn detection and GAPDH and CBB as loading control. The corresponding whole a-syn western blot can be found in Supplementary Figure 6A. **(B)** Quantification of a-syn signal intensity after normalization to loading control, shown relative to CTSD wt (*n* = 4–7). Statistical analysis was performed using a one-way ANOVA followed by a Tukey’s multiple comparison test. Significances were tested against CTSD wt (*p < 0.05). **(C)** Superimposed image of three CTSD wt structures post MDS. The active site residues are shown in red (spacefill) and amino acid A239 is shown in cyan (ball and stick). The loop carrying A239 is marked with a black arrow (filled) and the neighboring loop formed by amino acids 75–77 is marked with an arrow (black unfilled). **(D)** Superimposed image of three A239-CTSD structures post-MDS. Coloring and annotation with arrows as in **(C)**. Note the stronger deviation of the two loops when comparing to CTSD wt.

Since a-syn aggregation is a major hallmark of PD, and since CTSD is responsible for a-syn degradation within lysosomes, a-syn degradation properties of PD-linked CTSD variants were analyzed. Within SH-SY5Y CTSD KO cells, the effect of PD-CTSD variants on a-syn was studied after 72 h of overexpression by immunoblot analyses (Figure 4A; whole a-syn western blot: Supplementary Figure 6A). Remarkably, the CTSD variant A239V was able to decrease a-syn to a significantly higher degree compared to the CTSD wt (∼25% less a-syn; Figure 4B). Since all CTSD variants revealed similiar expression level as exhibted in the western blot (Figure 4A), this indicates that the point mutation A239V results in an enhanced a-syn proteolysis, being in line with higher enzymatic activity measured in SH-SY5Y CTSD KO and H4 CTSD KO cell lysates for this mutant (Figures 2E, 3C). Moreover, a-syn level increased after lysosomal (Bafilomycin A1) or CTSD (pepstatin A) inhibition in SH-SY5Y cells expressing CTSD wt (Supplementary Figure 6B). This indicates a crucial role of CTSD in a-syn homeostasis.

### Structural Analysis of CTSD A239V Variant

To gain structural insight into a possible mechanism responsible for this gain of activity of the CTSD A239V variant, molecular dynamics simulation (MDS) was applied. For this, the crystal structure of CTSD bound to the 2S4 inhibitor was used (Supplementary Figure 7A; PDB-ID: 4OBZ). As a start model for MDS, the inhibitor was removed (Supplementary Figure 7B) and alanine 239 was exchanged to a valine (UCSF Chimera; swapaa). Both, CTSD wt and CTSD A239V were simulated three times and largest movements were detected for two helices (indicated by arrows) that closed into the cavity formerly occupied by the inhibitor (Figures 4C,D). This movement was detected for both variants. Comparison of the three structures post-MDS revealed very little movement for CTSD wt in the loop carrying alanine 239 (filled black arrow) or the adjacent loop formed by amino acids 75–77 from the light chain (empty black arrow; Figure 4C). This was different for the A239V variant. Here, a larger deviation between the three resulting post-MDS structures was found (Figure 4D). A direct comparison of both variants from two different directions illustrates this difference in variability (Supplementary Figures 7E,F). Thus, insertion of valine at position 239 seems to destabilize not only the loop carrying the amino acid exchange itself, but also the neighboring loop formed by amino acids 75–77. This additional instability might also induce a higher flexibility and thereby accelerate substrate exchange rates.

**FIGURE 5:**
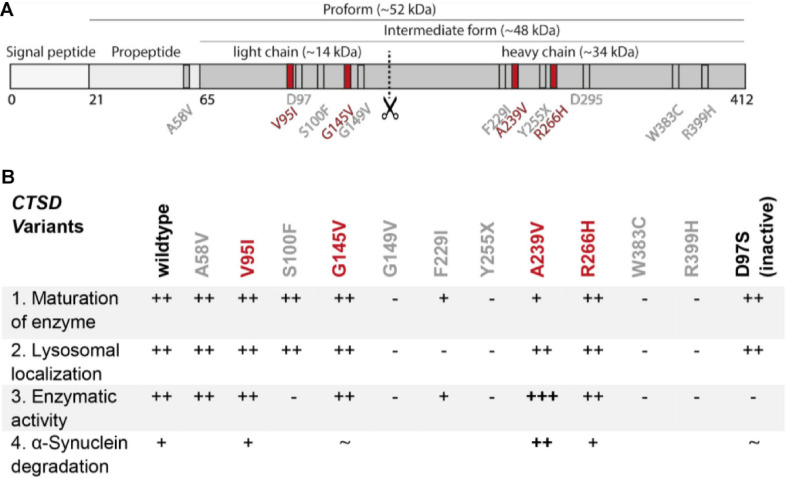
Summary figure of all in this study investigated disease-associated CTSD variants. **(A)** Schematic overview of localization of disease-associated CTSD variants. AD- and NCL-associated CTSD point mutation are shown in gray and PD-associated CTSD variants highlighted in red. Active site residues D97 and D295 are also depicted in gray. **(B)** Summary table of functional analyses of CTSD variants analyzed in this study. The CTSD wt is shown as reference and + and – symbols indicate deviation of each CSTD variant from the characteristic/behavior of CTSD wt within individual analyses: (1) maturation of enzyme (immunoblot analysis), (2) lysosomal localization (immunofluorescence analyses), (3) enzymatic activity (fluorogenic peptide cleavage assay), (4) a-syn degradation (overexpression and immunoblot analysis). The catalytically inactive CTSD variant (D97S) shows normal maturation and lysosomal localization, but no enzymatic activity. NCL- and AD-associated CTSD point mutations were not analyzed for a-syn degradation.

Overall, we found strong impairments in the enzymatic function of NCL-associated CTSD variants, but not in AD- and PD-linked mutants. These functional differences are surprising since mutations occur in the same gene and in a linear overview of the CTSD protein, NCL-/AD- and PD-mutations are found in close proximity (Figure 5A). Two regions within the light and heavy chain of the mature CTSD appear to be frequently affected by mutations: aa 95 – aa 149 (V95I, S100F, G145V, and G149V) and aa 229 – aa 266 (F229I, A239V, Y255X and R266H). This could indicate that both regions are crucial for proper CTSD function. As summarized in Figure 5B, NCL10-associated CTSD variants G149V, F229I, Y255X, W383C and R399H (all gray) do not mature, are less active and are not localized in the lysosome. Interestingly, the S100F mutant showed unimpaired maturation and lysosomal localization but no catalytic activity. The AD-associated variant A58V and PD-associated variants V95I, G145V, A239V, and R266H reach the lysosome and are enzymatically active. Surprisingly, the A239V mutant exhibited increased enzymatic activity, that also led to a higher a-syn turnover compared to the wt, as well as the other CTSD variants. By further analyzing this mutation, the structural properties of CTSD activation and substrate turnover can now be studied, helping in the design of therapeutic approaches targeting CTSD function.

## Discussion

To date, numerous studies emphasize the link between lysosomal dysfunction and neurodegenerative diseases (Nixon, 2013; Fraldi et al., 2016; Klein and Mazzulli, 2018; Zunke, 2020). The efficient degradation of lysosomal substrates (proteins, lipids, carbohydrates, etc.) is essential for neuronal survival. The aspartic protease CTSD is a major component of the lysosome and malfunction of the enzyme has been associated with severe neurodegenerative disorders, like the neuronal ceroid lipofuscinosis type 10 (NCL10), but also AD and PD (Vidoni et al., 2016).

In this study, we show that NCL10- and AD/PD-associated CTSD point mutants behaved completely different in comparable overexpression analyses. Whereas all NCL10-CTSD variants lack maturation and/or enzymatic activity, AD/PD-associated CTSD variants did not show major impairments in our experimental set-up. Corresponding to our immunofluorescence analysis, we speculate that NCL10-CTSD variants (except S100F) accumulate within the secretory pathway and do not reach lysosomal structures. The resulting absence of lysosomal CTSD corresponds to the severe pathology found in NCL10 patients (Siintola et al., 2006; Steinfeld et al., 2006; Fritchie et al., 2009; Hersheson et al., 2014; Kohlschutter et al., 2019; Regensburger et al., 2020). This course of disease is also partly recapitulated by the progressive phenotype of a CTSD-deficient mouse model, that dies prematurely at day 26 ± 1 (Saftig et al., 1995; Cullen et al., 2009; Marques et al., 2019).

In earlier studies, NCL10 patients were clinically examined and patient’s fibroblasts analyzed for CTSD maturation and activity. In Steinfeld et al. (2006), a compound heterozygous patient, carrying the biallelic missense mutations p.F229I (c.685T > A) and p.W383C (c.1149G > C), was reported with early blindness and progressive psychomotor retardation. Analysis of patient’s fibroblasts revealed less mature CTSD and a significant loss of enzymatic activity. Moreover, analyses of the individual point mutations in overexpression studies exhibited a significant decrease in mature CTSD in comparison to the wt for the F229I variant and absence of mature CTSD for the W383C variant (Steinfeld et al., 2006). This, together with a significant reduction of both CTSD variants in enzymatic activity, is in line with our data. Also, CTSD activity in fibroblasts of patients expressing the CTSD G149V as well as the R399H variant was found to be significantly reduced (Hersheson et al., 2014). Patients carrying the homozygous missense mutations p.G149V and p.R399H presented with ataxia, cognitive decline and retinitis pigmentosa at an age of 15 and 8 years, respectively (Hersheson et al., 2014). The homozygous mutations p.S100F (c.299C > T) and p.T255X (c.764dupA) resulted in premature death after 2 and 1 days after birth (Siintola et al., 2006; Fritchie et al., 2009). Interestingly, the CTSD S100F variant exhibited only marginal CTSD activity in patient fibroblasts, but the mature protein seemed to be stable in overexpression studies (Fritchie et al., 2009), which could both be confirmed in our study. Taken together, all here analyzed NCL10-CTSD variants show impaired CTSD maturation and/or enzymatic function, corresponding to the severe symptoms found in patients.

In contrast, the AD-associated A58V variant reached the lysosome and showed similar level of mature enzyme as well as comparable activity to the CTSD wt. In difference to all other here analyzed CTSD mutants, the A58V variant is located within the pro-peptide (A58V), which gets cleaved during maturation and is not present within the mature enzyme. This might explain why this CTSD variant does not show any cellular or functional impairments. In patients, CTSD A58V has been found to be associated with general intelligence in healthy older people (Payton et al., 2003). Moreover, AD patients expressing this CTSD mutation showed higher levels of amyloid β-protein as well as tau protein in the brain (Papassotiropoulos et al., 2002; Davidson et al., 2006; Riemenschneider et al., 2006). However, there is still controversy about the association and significant correlation between the expression of the A58V variant and the risk of developing AD [pro: (Papassotiropoulos et al., 2000; Mariani et al., 2006; Albayrak et al., 2010; Schuur et al., 2011; Sayad et al., 2014); contra: (Bagnoli et al., 2002; Mateo et al., 2002; Li et al., 2004; Mo et al., 2014)]. Nonetheless, CTSD has been discussed as therapeutic target in AD (Di Domenico et al., 2016).

Whereas clinical characterization of patients carrying NCL-/AD-associated CTSD mutations are available (Riemenschneider et al., 2006; Siintola et al., 2006; Steinfeld et al., 2006; Fritchie et al., 2009; Ehling et al., 2013; Hersheson et al., 2014), there is no clinical data on the here analyzed PD-associated CTSD variants, as they were found in a large genetic-meta analysis (Robak et al., 2017). PD is characterized by a-syn aggregation within the CNS. *In vitro* and *in vivo* mouse studies demonstrated that proteolysis of a-syn is mediated by the lysosomal protease CTSD (Sevlever et al., 2008) and vice versa, the loss of function of CTSD facilitates a-syn toxicity (Cullen et al., 2009). However, it is still not well-understood to what extend CTSD is involved in a-syn turnover in human neurons. Interestingly, it was shown recently that a-syn aggregates faster, creating a more toxic form under lysosomal pH conditions (Eymsh et al., 2020). This points to a critical role of CTSD in decreasing a-syn level within lysosomes by its degradative function.

In our study, the four PD-associated CTSD variants resulted in a similar maturation pattern and were correctly targeted to the lysosome, similar to CTSD wt in two different cell lines. Testing for a-syn degradation, we co-transfected cells with a-syn and the respective CTSD mutants. To our surprise, the proteolytic capacity of CTSD mutant A239V was enhanced ∼2.0 – 2.5-fold, which resulted in a more efficient clearance of the a-syn protein in comparison to the wt (∼25% less a-syn). For structural analyses, we utilized MDS and found structural changes within two loop regions that might be responsible for the increase in activity found in CTSD A239V. The higher flexibility of this region was deduced from the different conformations received after MDS. Enzyme activity is always balanced between flexibility to interact with the substrate and rigidity to ensure stability of the enzyme (Shoichet et al., 1995). For NADH oxidase of *Thermus thermophilus* with a temperature optimum around 70°C, induction of flexibility can also be achieved by the addition of 1.0–1.3 M urea at ambient temperature (Zoldak et al., 2003). In our case, the insertion of a slightly larger amino acid (alanine to valine) might induce this bit of flexibility, that accounts for the increase in activity. Interestingly, this mutation was expressed to a reduced extent than other here analyzed disease-associated CTSD variants. The increased flexibility of the loop might also result in a less stable protein that is more prone to degradation. To further validate the role of the two loops within the CTSD protein, additional experiments using recombinant enzyme have to be carried out. Nonetheless, this opens novel possibilities for treatment strategies targeting protein aggregation in neurodegenerative disorders. Hence, the use of recombinant CTSD as therapeutic approach has been shown by an enzyme replacement approach within a mouse model lacking CTSD. Marques et al. (2019) provide evidence that the treatment with recombinant CTSD corrects defective proteolysis and autophagy in a murine NCL10 model.

Nevertheless, the question about the role and impact of CTSD mutations on the pathology of neurodegenerative diseases is still elusive. Since AD- and PD-associated CTSD variants did not exhibit major impairments in maturation and enzymatic activity in our overexpression analyses, it could be the case that our experimental set-up is not sensitive enough to pick up small functional changes. Since AD and PD usually present at higher ages, post-mitotic neuronal cells might enrich small amounts of accumulating substrates over time until reaching critical concentrations causing neurotoxicity, neuronal cell death and pathology (Hara et al., 2006; Komatsu et al., 2006). Moreover, it is possible that other lysosomal cathepsins, which have also been shown to be involved in lysosomal a-syn degradation (CTSB, CTSL) (McGlinchey and Lee, 2015; McGlinchey et al., 2019), are able to compensate for CTSD deficiencies.

The role of the more active CTSD A239V mutant seems -on first glance- difficult to explain. However, a recent study by McGlinchey et al. (2019) showed that C-terminal a-syn truncations are linked to the enzymatic activity of lysosomal cathepsins. Importantly, these C-terminally truncated a-syn forms have been shown to promote aggregation and fibril formation (Crowther et al., 1998; Hoyer et al., 2004; Levitan et al., 2011; van der Wateren et al., 2018). In this regard, CSTD has also been shown to generate C-terminally truncated a-syn variants (McGlinchey and Lee, 2015). This might explain why this more active CTSD variant potentially drives a-syn aggregation and disease pathology. In a follow-up study, this needs to be further validated by *in vitro* studies analyzing the cleavage fragments of a-syn after incubation with the disease-associated CTSD variant. Moreover, it is also possible that other concomitant genomic variants represent the primary cause of disease, and that CTSD mutations are “only” supporting, but not the cause of disease pathology. This might also be the case for PD patients carrying the CTSD A239V mutation. Here, follow-up studies are needed to examine the patients for further genetic variations. For instance, some AD patients carrying the CSTD A58V variant were also affected by a mutation within Presenilin 1, which is known to be associated with early onset AD (Ehling et al., 2013). To analyze the effect of each individual CTSD point mutation in disease, analyses of CTSD homeostasis within patient material (e.g., fibroblasts) should be performed in future studies.

Summarizing, our study sheds light into the structure and regulation of enzymatic CTSD function as well as a-syn degradation. This better understanding might enable us in the future to design treatment strategies targeting CTSD function in order to reduce abnormal protein aggregation in PD and other neurodegenerative disorders.

## Data Availability Statement

The original contributions presented in the study are included in the article/Supplementary Material, further inquiries can be directed to the corresponding author.

## Author Contributions

FZ: conceptualization and supervision. JB, SPH, AD, LW, and PA: experiments. JB, SPH, AD, and FZ: writing – original draft preparation. JB and FZ: writing – revision. JB, JD, PA, and FZ: visualization. SR-J, PA, and FZ: funding acquisition. All authors contributed to the article and approved the submitted version.

## Conflict of Interest

The authors declare that the research was conducted in the absence of any commercial or financial relationships that could be construed as a potential conflict of interest.

## Supplementary Material

Supplementary Figure 1 Generating CTSD deficient cell lines (SH-SY5Y and H4). **(A)** A CRISPR/Cas9 RNP (Ribonucleoprotein) approach by using three guide RNA (gRNA, green arrows and sequence) targeting the *CTSD* gene at exon 2 was used. Sanger sequencing analysis from CTSD wt (same sequence for SH-SY5Y and H4 cells), SH-SY5Y CTSD KO clone 10 (Cl. 10) and H4 CTSD KO clone 2 (Cl. 2) is shown by the corresponding chromatogram of the *CTSD* exon 2 gene. RNP-mediated editing resulted in a deletion of 91bp for exon 2 in SH-SY5Y cells (Cl.10) and a deletion of 106 bp for exon 2 in H4 cells (Cl. 2). **(B)** Representative immunoblot of different cell systems comparing endogenous CTSD levels. **(C)** Confocal microscopy images of CTSD (green) and lysosomal marker [LAMP2 (red)] in SH-SY5Y cells deficient for CTSD (CTSD KO), CTSD KO reconstituted with CTSD wt and wt cells exhibiting endogenous CTSD. Analysis of CTSD signal intensity indicates non-significant (n.s.) higher CTSD level after overexpression in SH-SY5Y cells in comparison to endogenous CTSD level within SH-SY5Y wt cells (*n* = 15 cells). Scale bar: 20 μm. Statistical analyses were performed by using a one-way ANOVA followed by a Tukey’s multiple comparison test.

Supplementary Figure 2 Cellular localization of AD-/NCL-associated CTSD variants in SH-SY5Y CTSD KO cells. Representative confocal microscopy images of immunofluorescence stainings of CTSD variants overexpressed in SH-SY5Y CTSD KO cells. Cells were stained for CTSD (green), the lysosomal marker LAMP2 (red) and DAPI as nuclear stain (blue). Yellow staining indicates lysosomal localization of CTSD variant. Scale bar: 20 μm.

Supplementary Figure 3 Analyses of confocal images for CTSD localization, lysosomal vesicle number and vesicle size. **(A)** Analysis of intracellular co-localization of AD-/NCL-associated CTSD variants in SH-SY5Y cells deficient for CTSD (CTSD KO). The Pearson’s correlation coefficient was used to determine the co-localization of AD-/NCL-CTSD mutants with lysosomal marker LAMP2 (*n* = 11–15, derived from cell from three independent experiments). **(B)** Quantification of LAMP2-positive vesicle number per cell (*n* = 11–19, derived from three independent experiments). **(C)** Analyses of average LAMP2-positive vesicle size per cell (*n* = 12–19, derived from three independent experiments). All statistical analyses were performed by using a one-way ANOVA followed by a Tukey’s multiple comparison test. *^∗^p* < 0.05, *^∗∗∗^p* < 0.0001, ^****^*p* < 0.0001, n.s., not significant in comparison to the wt.

Supplementary Figure 4 Cellular localization of PD-associated CTSD variants in SH-SY5Y CTSD KO cells. Representative confocal microscopy images of immunofluorescence stainings of PD-CTSD variants, overexpressed in SH-SY5Y cells deficient for CTSD (CTSD KO). Cells were stained for CTSD (green), the lysosomal marker LAMP2 (red), and DAPI as nuclear stain (blue). Yellow staining indicates lysosomal localization of CTSD variant. Scale bar: 20 μm.

Supplementary Figure 5 Analyses of confocal images for CTSD localization, lysosomal vesicle number, and size as well as maturation of PD-associated CTSD variants. **(A)** Analysis of intracellular co-localization of PD-associated CTSD variants in SH-SY5Y cells deficient for CTSD (CTSD KO). The Pearson’s correlation coefficient was used to determine the co-localization of PD-CTSD mutants with lysosomal marker LAMP2 (total *n* = 11–15 in three independent experiments). **(B)** Quantification of LAMP2-positive vesicle number per cell (*n* = 11–19, derived from cells from three independent experiments). **(C)** Analyses of average LAMP2-positive vesicle size per cell (*n* = 12–19, derived from three independent experiments). **(D,E)** Analysis of protein maturation of PD-CTSD mutants: ratio of mature CTSD divided by immature CTSD level in SH-SY5Y CTSD KO cells **(D)** and H4 CTSD KO cells **(E)** both normalized to CTSD wt (*n* = 4–8). All statistical analyses were performed by using a one-way ANOVA followed by a Tukey’s multiple comparison test. ^∗∗^*p* < 0.01, ^****^*p* < 0.0001.

Supplementary Figure 6 a-Syn western blot and level after lysosomal inhibition. **(A)** Whole immunoblot of Figure 4A exhibiting no a-syn bands at higher molecular weight (a-syn antibody: Syn-1). **(B)** Representative immunoblot of SH-SY5Y CTSD KO cells transfected with CTSD wt co-expressing a-syn. Cells were treated with lysosomal inhibitor bafilomycin A1 (BafA1) or cathepsin D inhibitor pepstatin A (PepA), resulting in increased a-syn level (a-syn antibody: Syn-1). Analysis of a-syn signal after normalization to loading control indicating a significant increase of a-syn level after treatment of cells with BafA1 and PepA. Statistical analysis was performed using a one-way ANOVA followed by a multiple comparison test. Significances were tested against CTSD mock (*^∗^p* < 0.05, ^∗∗^*p* < 0.01).

Supplementary Figure 7 Molecular dynamics simulation (MDS) of the A239V variant of CTSD. **(A)** Original crystal structure of CTSD (PDB-ID: 4OBZ) bound to the 2S4 inhibitor. The light chain is displayed in blue, the heavy chain in gray. Active site residues are shown in spacefill red with 2S4 in orange (ball and stick). Alanine 239 is shown in cyan (ball and stick). **(B)** As a start conformation for MDS, the 2S4 inhibitor was removed to allow conversion of the structure into an unbound state. The single amino acid exchange was introduced using UCSF Chimera (swapaa). Coloring as in **(A)**. **(C)** Superimposed image of the start conformation (coloring as in **A**) and the three end conformations post-MDS (green) for CTSD wt. **(D)** Superimposed image of the start conformation (coloring as in **A**) and the three end conformations post-MDS (orange) for A239V-CTSD. **(E)** Superimposed image of the three end-conformations post-MDS for CTSD wt (green) and CTSD A239V (orange). The insert shows the loop carrying the A239V variant (filled black arrow) and the loop formed by amino acids 75–77 located within the light chain (empty black arrow). **(F)** As in **(E)**, but with the structures turned by 90° around the *y*-axis.
