# Corrigendum: Cathepsin D Variants Associated With Neurodegenerative Diseases Show Dysregulated Functionality and Modified α-Synuclein Degradation Properties

**DOI:** 10.3389/fcell.2021.671532

**Published:** 2021-06-23

**Authors:** Josina Bunk, Susy Prieto Huarcaya, Alice Drobny, Jan Philipp Dobert, Lina Walther, Stefan Rose-John, Philipp Arnold, Friederike Zunke

**Affiliations:** ^1^Institute of Biochemistry, Christian-Albrechts-Universität zu Kiel, Kiel, Germany; ^2^Department of Molecular Neurology, University Hospital Erlangen, Friedrich-Alexander-Universität Erlangen-Nürnberg, Erlangen, Germany; ^3^Institute of Anatomy, Christian-Albrechts-Universität zu Kiel, Kiel, Germany; ^4^Institute of Anatomy, Functional and Clinical Anatomy, Friedrich-Alexander-University Erlangen-Nürnberg, Erlangen, Germany

**Keywords:** lysosomal degradation, molecular dynamics simulation, Parkinson's disease, neuronal ceroid lipofuscinoses, lysosomes, alpha-synuclein, cathepsin D

In the original article, there was a mistake in Figure 1C as published. In the Coomassie brilliant blue (CBB) protein staining, which was used as additional loading control (besides GAPDH), 11 instead of 10 sample lanes (+ protein ladder) were shown in the original Figure 1C. This mistake resulted from an additional sample (transfection control), that was loaded on the far right. This lane was not shown in both immunoblots (CTSD and GAPDH), but accidentally was still shown in the CBB control. The corrected Figure 1C appears below.

**Figure 1:**
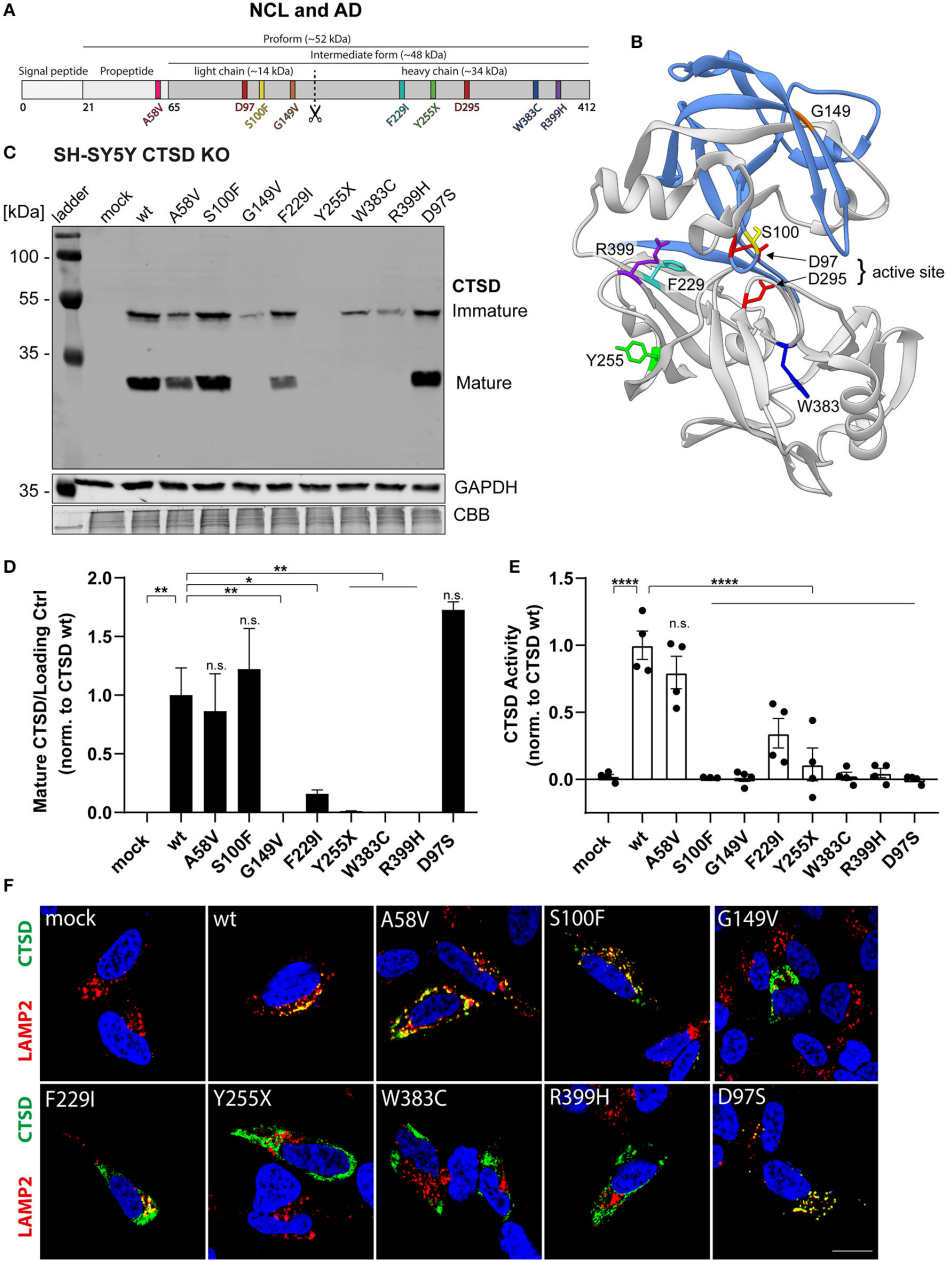
Characterization of CTSD variants associated with neurodegenerative diseases in SH-SY5Y CTSD KO cells. **(A)** Schematic overview of NCL- and AD-associated CTSD variants analyzed in this study. During protein maturation, the signal peptide (20 aa) and the propeptide (44 aa) are removed, generating an intermediate form (~48 kDa) that is further processed in the lysosome into a double-chain mature form comprised of a light chain (~14 kDa) and a heavy chain (~34 kDa; symbolized by scissor symbol). Both chains remain associated by hydrophobic interactions. Point mutations found in NCL- and AD- patients located within different protein parts are shown in different colors (A58V, pink, propeptide; S100F, yellow, light chain; G149V, orange, light chain; F229I, light blue, heavy chain; Y255X, green, heavy chain; W383C, dark blue, heavy chain; R399H, purple, heavy chain). Aspartates D97 and D295 as part the catalytic site are highlighted in red. **(B)** Crystal structure model of mature CTSD consisting of the light chain (blue) and the heavy chain (gray) [PDB-ID: 4OBZ (Gradler et al., 2014)]. The active site, consisting of the two aspartates D97 and D295, is shown in red. Other colors indicate disease-associated point mutations within the CTSD protein (same color code as in A). **(C)** Representative immunoblot of transiently overexpressed CTSD wildtype (wt) and NCL-/AD-associated CTSD variants, as well as enzymatically inactive control (D97S) in SH-SY5Y CTSD KO cells. An anti-CTSD antibody was used for the detection of immature (pro- and intermediate form) as well as mature CTSD (heavy chain, 34 kDa). GAPDH and coomassie brilliant blue (CBB) were used as a loading control. **(D)** Quantification of western blot signal intensity of mature CTSD (heavy chain) normalized to GAPDH and expressed relative to CTSD wt (n = 4). **(E)** Analysis of CTSD activity assessed in whole cell lysates utilizing a fluorogenic CTSD peptide cleavage assay. The activity was normalized to CTSD wt (n = 4). **(F)** Representative immunofluorescence pictures of SH-SY5Y CTSD KO cells expressing CTSD wt or NCL-/AD-associated variants. Cells were visualized by staining of CTSD (green), the lysosomal associated membrane protein LAMP2 (red), and DAPI as nuclear staining (blue). Scale bar: 20 mm. Confocal images showing the single channels can be found in Supplementary Figure 2. All statistical analyses were performed using a one-way ANOVA followed by a Tukey's multiple comparison test. *p < 0.05, **p < 0.01, ****p < 0.0001, n.s., not significant in comparison to wt.

The authors apologize for this error and state that this does not change the scientific conclusions of the article in any way. The original article has been updated.
