# Supplementary figures and images for: Cathepsin D Variants Associated With Neurodegenerative Diseases Show Dysregulated Functionality and Modified α-Synuclein Degradation Properties
Source: Front Cell Dev Biol. 2021 Feb 11;9:581805. doi: 10.3389/fcell.2021.581805 (PMC7928348; doi:10.3389/fcell.2021.581805)

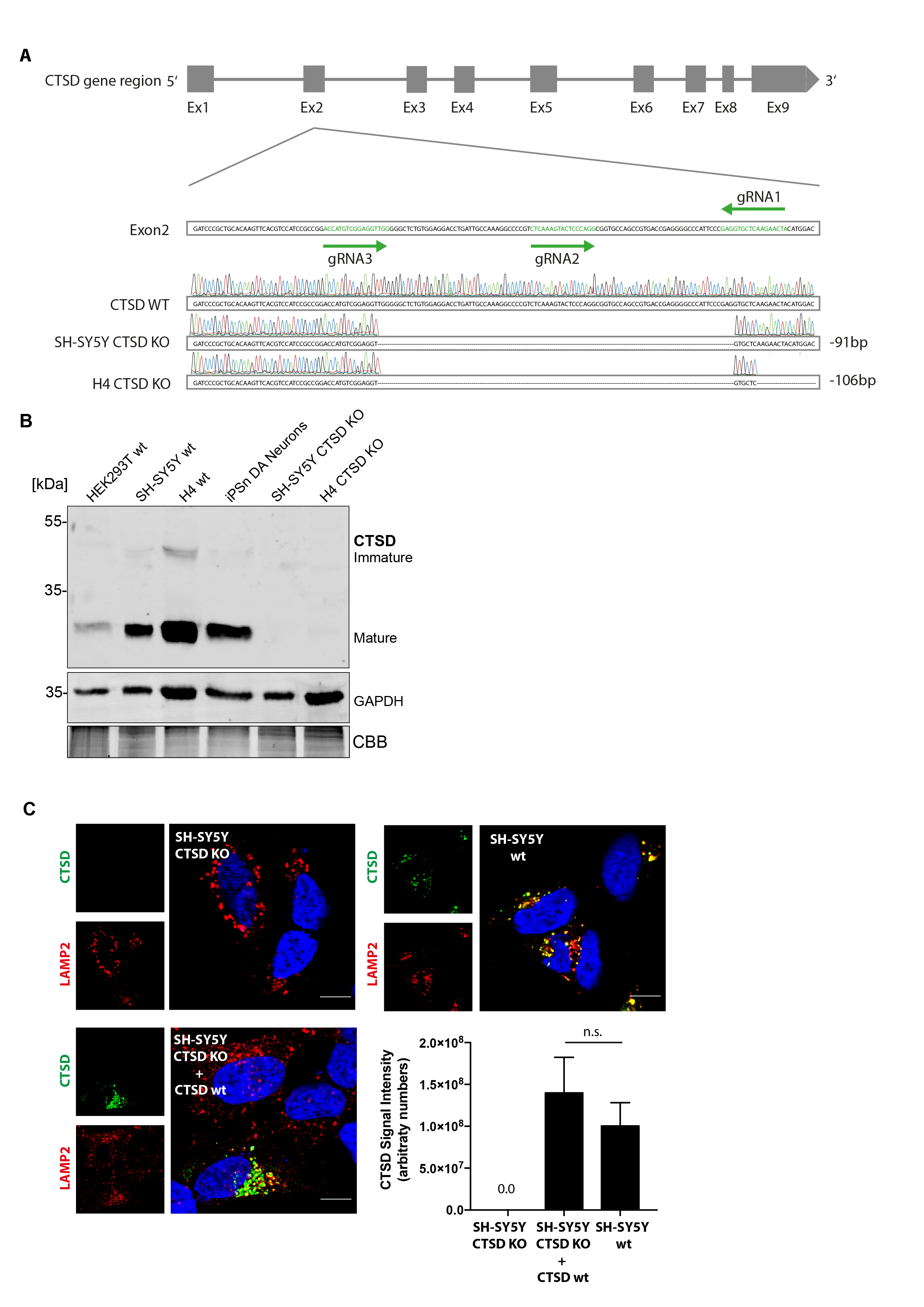

Supplement: Supplementary Figure 1 — Generating CTSD deficient cell lines (SH-SY5Y and H4). (A) A CRISPR/Cas9 RNP (Ribonucleoprotein) approach by using three guide RNA (gRNA, green arrows and sequence) targeting the CTSD gene at exon 2 was used. Sanger sequencing analysis from CTSD wt (same sequence for SH-SY5Y and H4 cells), SH-SY5Y CTSD KO clone 10 (Cl. 10) and H4 CTSD KO clone 2 (Cl. 2) is shown by the corresponding chromatogram of the CTSD exon 2 gene. RNP-mediated editing resulted in a deletion of 91bp for exon 2 in SH-SY5Y cells (Cl.10) and a deletion of 106 bp for exon 2 in H4 cells (Cl. 2). (B) Representative immunoblot of different cell systems comparing endogenous CTSD levels. (C) Confocal microscopy images of CTSD (green) and lysosomal marker [LAMP2 (red)] in SH-SY5Y cells deficient for CTSD (CTSD KO), CTSD KO reconstituted with CTSD wt and wt cells exhibiting endogenous CTSD. Analysis of CTSD signal intensity indicates non-significant (n.s.) higher CTSD level after overexpression in SH-SY5Y cells in comparison to endogenous CTSD level within SH-SY5Y wt cells (n = 15 cells). Scale bar: 20 μm. Statistical analyses were performed by using a one-way ANOVA followed by a Tukey’s multiple comparison test. [file Image_1.png]

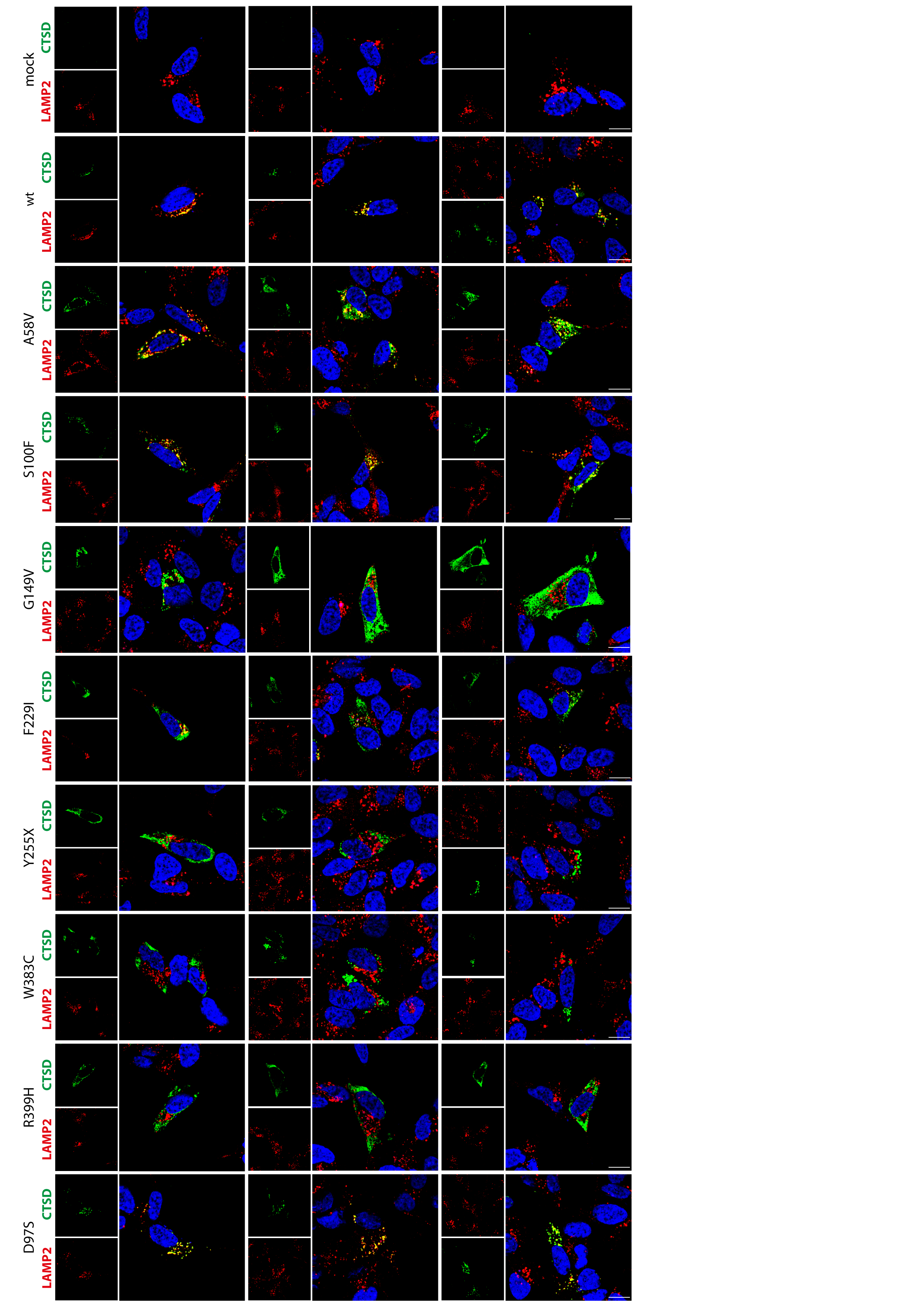

Supplement: Supplementary Figure 2 — Cellular localization of AD-/NCL-associated CTSD variants in SH-SY5Y CTSD KO cells. Representative confocal microscopy images of immunofluorescence stainings of CTSD variants overexpressed in SH-SY5Y CTSD KO cells. Cells were stained for CTSD (green), the lysosomal marker LAMP2 (red) and DAPI as nuclear stain (blue). Yellow staining indicates lysosomal localization of CTSD variant. Scale bar: 20 μm. [file Image_2.TIF]

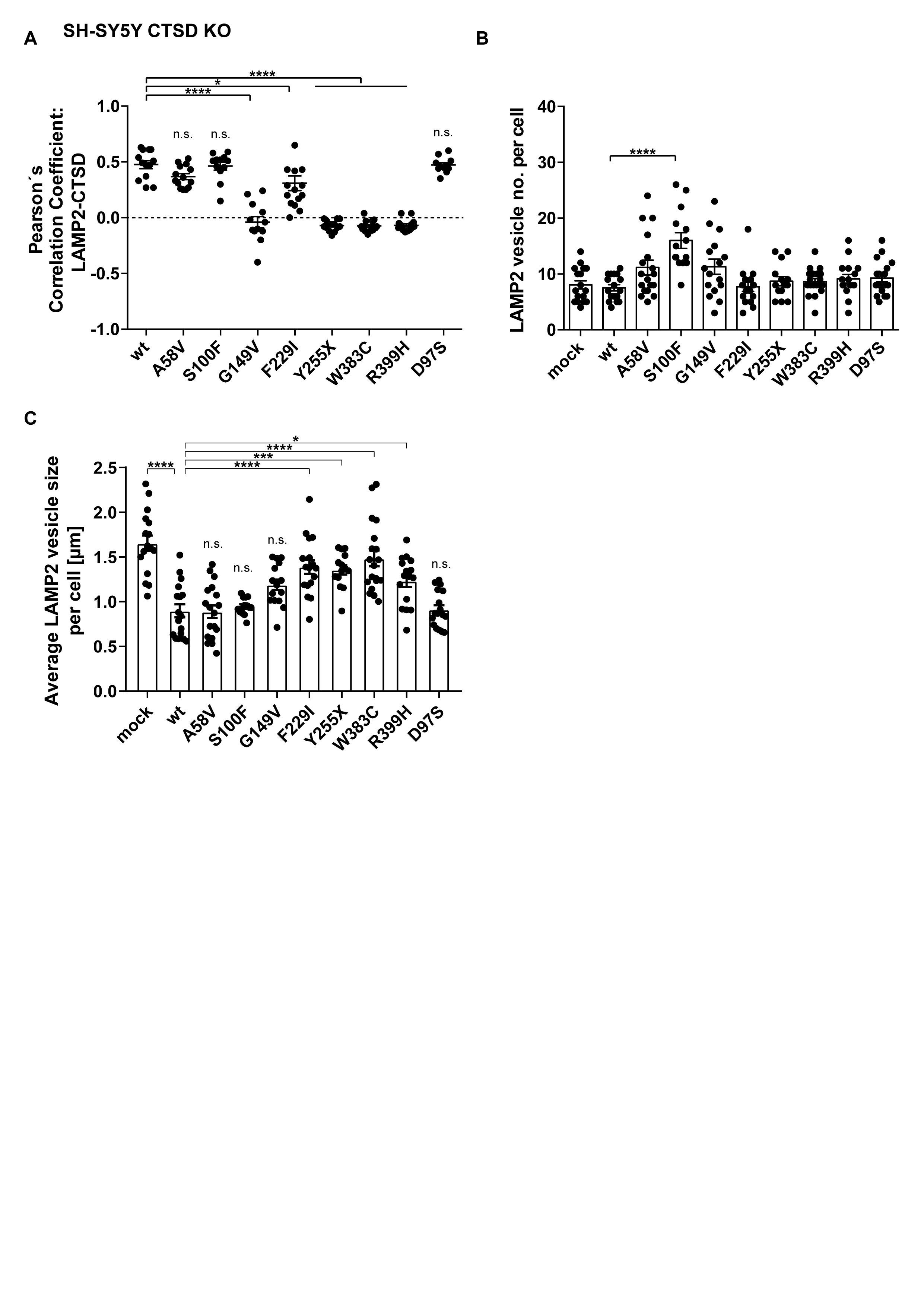

Supplement: Supplementary Figure 3 — Analyses of confocal images for CTSD localization, lysosomal vesicle number and vesicle size. (A) Analysis of intracellular co-localization of AD-/NCL-associated CTSD variants in SH-SY5Y cells deficient for CTSD (CTSD KO). The Pearson’s correlation coefficient was used to determine the co-localization of AD-/NCL-CTSD mutants with lysosomal marker LAMP2 (n = 11–15, derived from cell from three independent experiments). (B) Quantification of LAMP2-positive vesicle number per cell (n = 11–19, derived from three independent experiments). (C) Analyses of average LAMP2-positive vesicle size per cell (n = 12–19, derived from three independent experiments). All statistical analyses were performed by using a one-way ANOVA followed by a Tukey’s multiple comparison test. ∗p < 0.05, ∗∗∗p < 0.0001, ****p < 0.0001, n.s., not significant in comparison to the wt. [file Image_3.TIF]

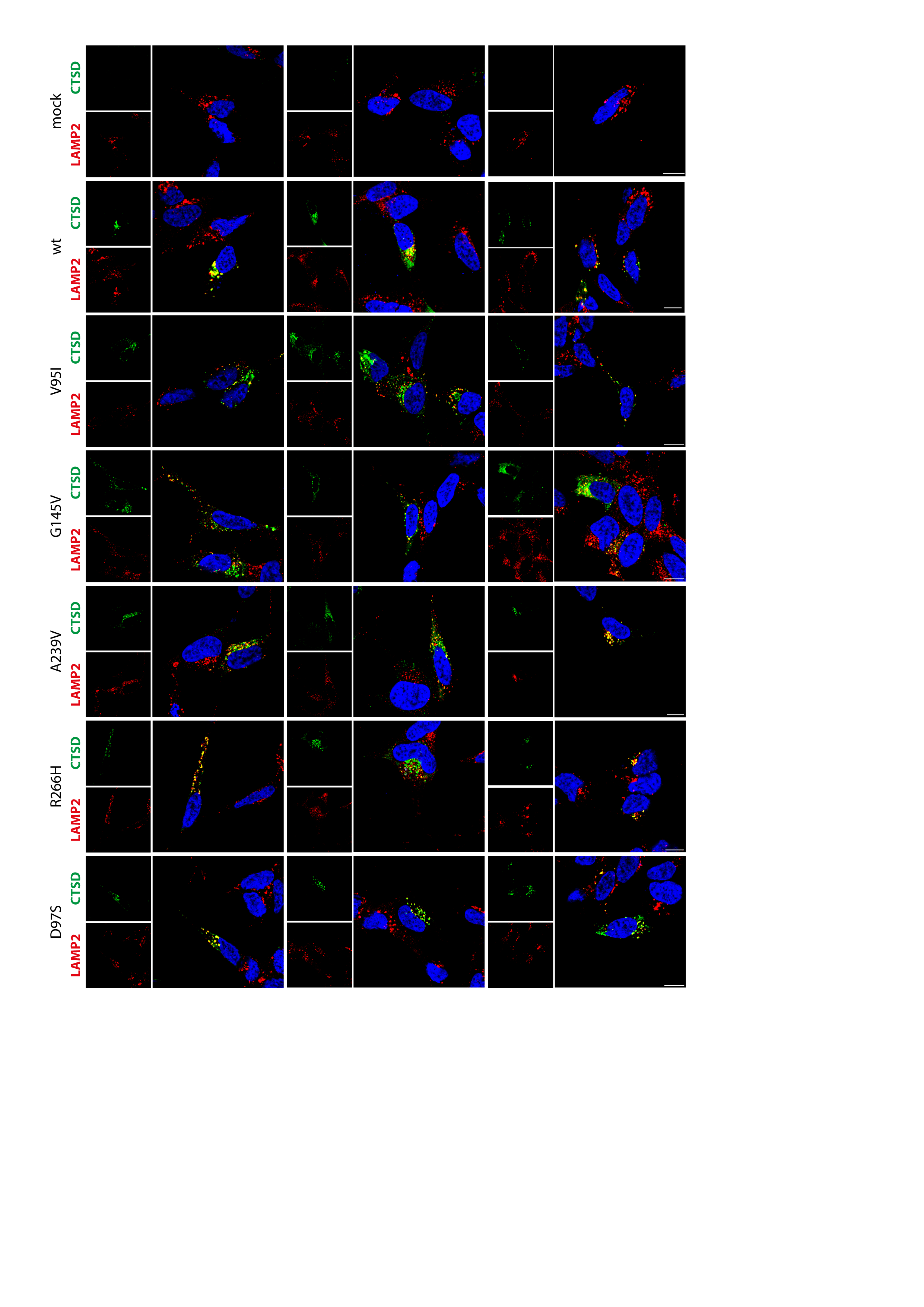

Supplement: Supplementary Figure 4 — Cellular localization of PD-associated CTSD variants in SH-SY5Y CTSD KO cells. Representative confocal microscopy images of immunofluorescence stainings of PD-CTSD variants, overexpressed in SH-SY5Y cells deficient for CTSD (CTSD KO). Cells were stained for CTSD (green), the lysosomal marker LAMP2 (red), and DAPI as nuclear stain (blue). Yellow staining indicates lysosomal localization of CTSD variant. Scale bar: 20 μm. [file Image_4.TIF]

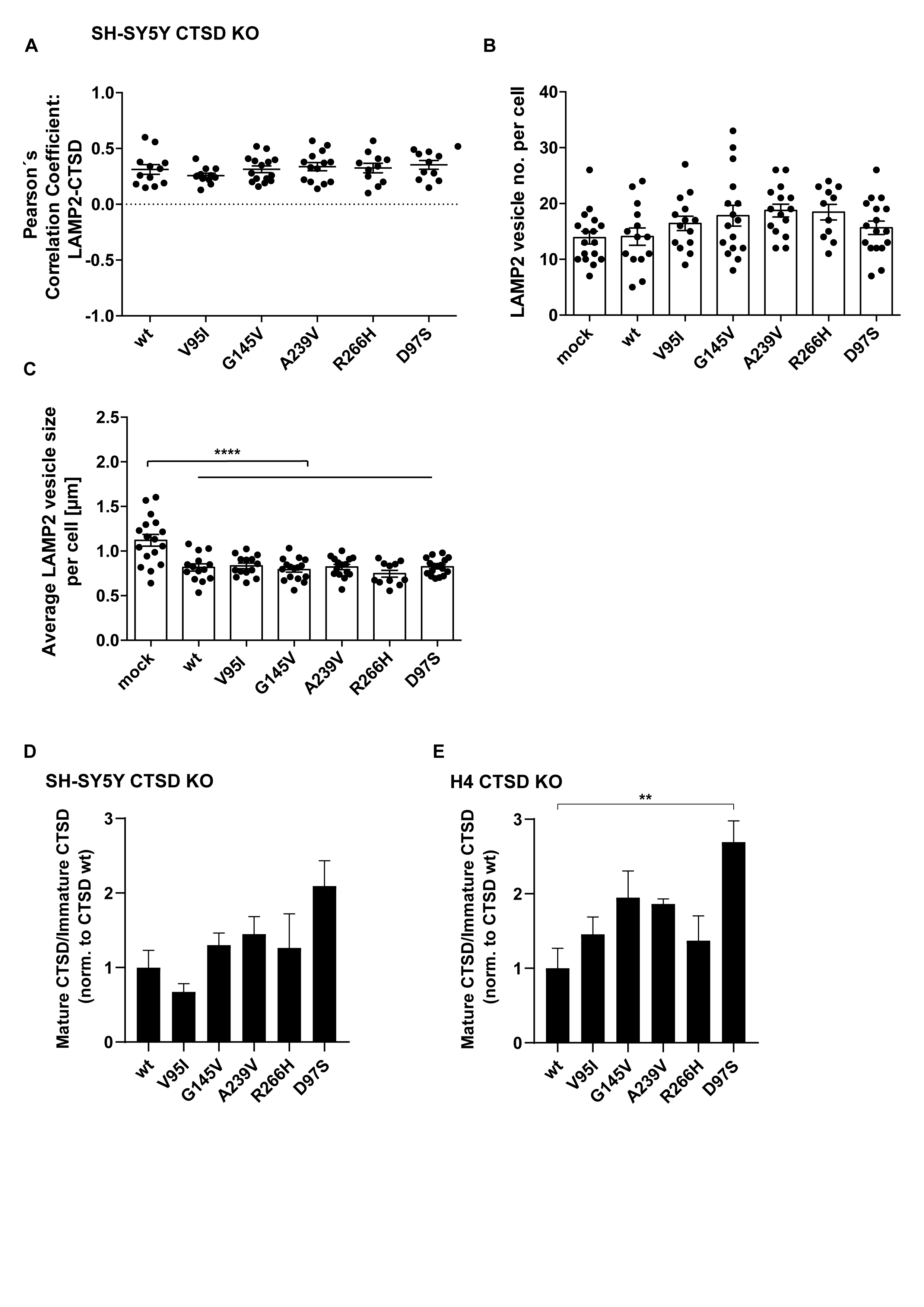

Supplement: Supplementary Figure 5 — Analyses of confocal images for CTSD localization, lysosomal vesicle number, and size as well as maturation of PD-associated CTSD variants. (A) Analysis of intracellular co-localization of PD-associated CTSD variants in SH-SY5Y cells deficient for CTSD (CTSD KO). The Pearson’s correlation coefficient was used to determine the co-localization of PD-CTSD mutants with lysosomal marker LAMP2 (total n = 11–15 in three independent experiments). (B) Quantification of LAMP2-positive vesicle number per cell (n = 11–19, derived from cells from three independent experiments). (C) Analyses of average LAMP2-positive vesicle size per cell (n = 12–19, derived from three independent experiments). (D,E) Analysis of protein maturation of PD-CTSD mutants: ratio of mature CTSD divided by immature CTSD level in SH-SY5Y CTSD KO cells (D) and H4 CTSD KO cells (E) both normalized to CTSD wt (n = 4–8). All statistical analyses were performed by using a one-way ANOVA followed by a Tukey’s multiple comparison test. ∗∗p < 0.01, ****p < 0.0001. [file Image_5.tif]

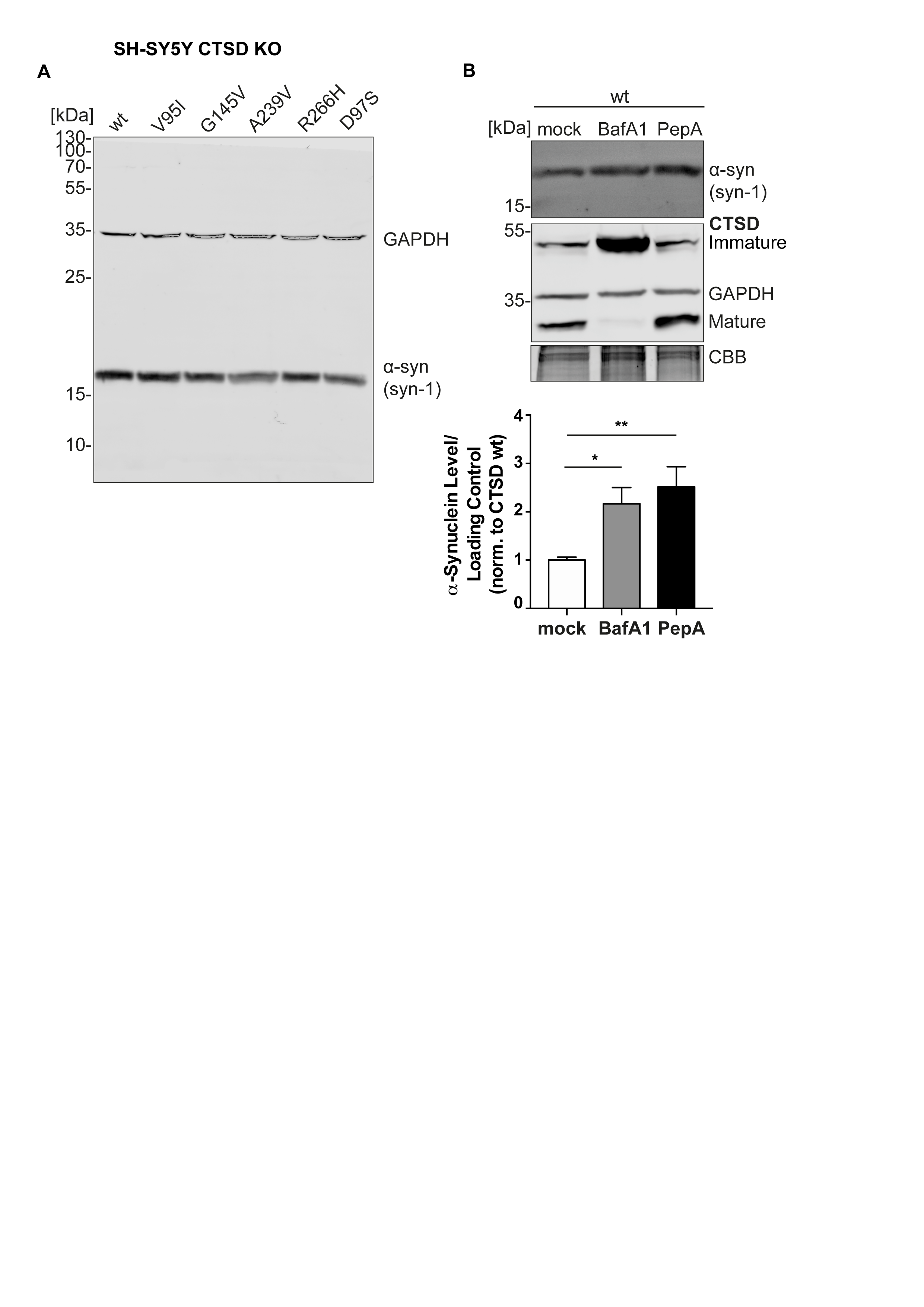

Supplement: Supplementary Figure 6 — a-Syn western blot and level after lysosomal inhibition. (A) Whole immunoblot of Figure 4A exhibiting no a-syn bands at higher molecular weight (a-syn antibody: Syn-1). (B) Representative immunoblot of SH-SY5Y CTSD KO cells transfected with CTSD wt co-expressing a-syn. Cells were treated with lysosomal inhibitor bafilomycin A1 (BafA1) or cathepsin D inhibitor pepstatin A (PepA), resulting in increased a-syn level (a-syn antibody: Syn-1). Analysis of a-syn signal after normalization to loading control indicating a significant increase of a-syn level after treatment of cells with BafA1 and PepA. Statistical analysis was performed using a one-way ANOVA followed by a multiple comparison test. Significances were tested against CTSD mock (∗p < 0.05, ∗∗p < 0.01). [file Image_6.tif]

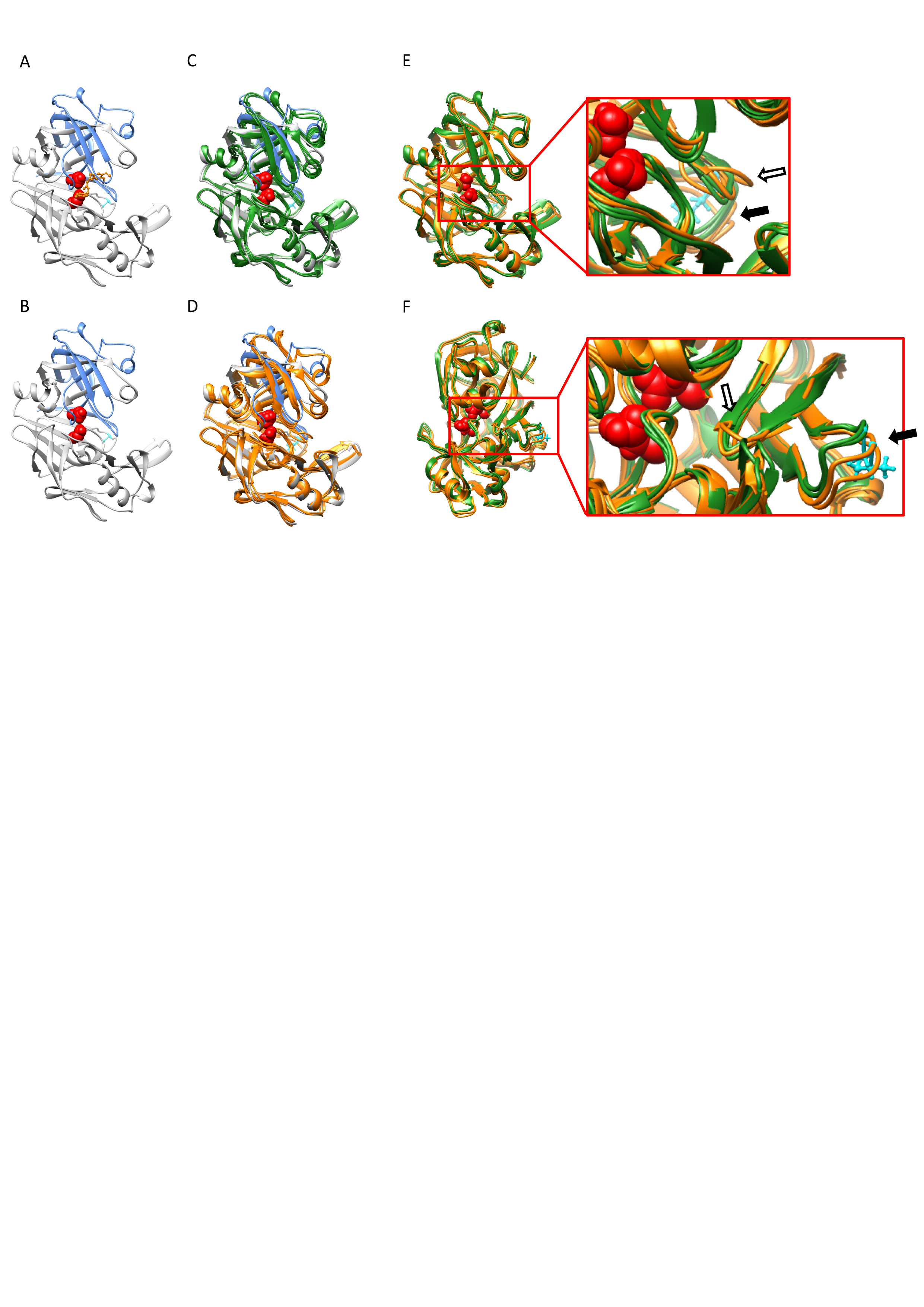

Supplement: Supplementary Figure 7 — Molecular dynamics simulation (MDS) of the A239V variant of CTSD. (A) Original crystal structure of CTSD (PDB-ID: 4OBZ) bound to the 2S4 inhibitor. The light chain is displayed in blue, the heavy chain in gray. Active site residues are shown in spacefill red with 2S4 in orange (ball and stick). Alanine 239 is shown in cyan (ball and stick). (B) As a start conformation for MDS, the 2S4 inhibitor was removed to allow conversion of the structure into an unbound state. The single amino acid exchange was introduced using UCSF Chimera (swapaa). Coloring as in (A). (C) Superimposed image of the start conformation (coloring as in A) and the three end conformations post-MDS (green) for CTSD wt. (D) Superimposed image of the start conformation (coloring as in A) and the three end conformations post-MDS (orange) for A239V-CTSD. (E) Superimposed image of the three end-conformations post-MDS for CTSD wt (green) and CTSD A239V (orange). The insert shows the loop carrying the A239V variant (filled black arrow) and the loop formed by amino acids 75–77 located within the light chain (empty black arrow). (F) As in (E), but with the structures turned by 90° around the y-axis. [file Image_7.tif]
