# Supplementary material for: Cathepsin D Variants Associated With Neurodegenerative Diseases Show Dysregulated Functionality and Modified α-Synuclein Degradation Properties
Source: Front Cell Dev Biol. 2021 Feb 11;9:581805. doi: 10.3389/fcell.2021.581805 (PMC7928348; doi:10.3389/fcell.2021.581805)
Supplement: Supplementary file 8 [file Data_Sheet_1.PDF]

**Fig. 1D. NCL Mature Cathepsin D/ Loading Control (norm. CTSD wt), SHS-S5Y5 CTSD KO cells**

| | |
|---|---|
| Number of families | 1 |
| Number of comparisons per family | 45 |
| Alpha | 0,05 |

| Tukey's multiple comparisons test | Mean Diff, | 95,00% CI of diff, | Significant? | Summary | Adjusted P Value | |
|---|---|---|---|---|---|---|
| mock vs. wt | -0,999 | -1,743 to -0,2548 | Yes | ** | 0,0028 | A-B |
| mock vs. A58V | -0,8626 | -1,607 to -0,1184 | Yes | * | 0,0137 | A-C |
| mock vs. S100F | -1,222 | -2,026 to -0,4178 | Yes | *** | 0,0006 | A-D |
| mock vs. G149V | -0,00114 | -0,7454 to 0,7431 | No | ns | >0,9999 | A-E |
| mock vs. F229I | -0,1575 | -0,9017 to 0,5868 | No | ns | 0,9991 | A-F |
| mock vs. Y255X | -0,01097 | -0,7552 to 0,7333 | No | ns | >0,9999 | A-G |
| mock vs. W383C | -0,001438 | -0,7457 to 0,7428 | No | ns | >0,9999 | A-H |
| mock vs. R399H | -0,0004265 | -0,7447 to 0,7438 | No | ns | >0,9999 | A-I |
| mock vs. D97S | -1,726 | -2,530 to -0,9221 | Yes | **** | <0,0001 | A-J |
| wt vs. A58V | 0,1364 | -0,6078 to 0,8807 | No | ns | 0,9997 | B-C |
| wt vs. S100F | -0,2227 | -1,027 to 0,5812 | No | ns | 0,9929 | B-D |
| wt vs. G149V | 0,9979 | 0,2537 to 1,742 | Yes | ** | 0,0028 | B-E |
| wt vs. F229I | 0,8416 | 0,09733 to 1,586 | Yes | * | 0,0174 | B-F |
| wt vs. Y255X | 0,9881 | 0,2438 to 1,732 | Yes | ** | 0,0032 | B-G |
| wt vs. W383C | 0,9976 | 0,2534 to 1,742 | Yes | ** | 0,0028 | B-H |
| wt vs. R399H | 0,9986 | 0,2544 to 1,743 | Yes | ** | 0,0028 | B-I |
| wt vs. D97S | -0,7269 | -1,531 to 0,07698 | No | ns | 0,102 | B-J |
| A58V vs. S100F | -0,3591 | -1,163 to 0,4448 | No | ns | 0,8678 | C-D |
| A58V vs. G149V | 0,8615 | 0,1172 to 1,606 | Yes | * | 0,0139 | C-E |
| A58V vs. F229I | 0,7051 | -0,03912 to 1,449 | No | ns | 0,0745 | C-F |
| A58V vs. Y255X | 0,8516 | 0,1074 to 1,596 | Yes | * | 0,0155 | C-G |
| A58V vs. W383C | 0,8612 | 0,1169 to 1,605 | Yes | * | 0,014 | C-H |
| A58V vs. R399H | 0,8622 | 0,1179 to 1,606 | Yes | * | 0,0138 | C-I |
| A58V vs. D97S | -0,8633 | -1,667 to -0,05947 | Yes | * | 0,0278 | C-J |
| S100F vs. G149V | 1,221 | 0,4167 to 2,024 | Yes | *** | 0,0006 | D-E |
| S100F vs. F229I | 1,064 | 0,2604 to 1,868 | Yes | ** | 0,0033 | D-F |
| S100F vs. Y255X | 1,211 | 0,4069 to 2,015 | Yes | *** | 0,0006 | D-G |
| S100F vs. W383C | 1,22 | 0,4164 to 2,024 | Yes | *** | 0,0006 | D-H |
| S100F vs. R399H | 1,221 | 0,4174 to 2,025 | Yes | *** | 0,0006 | D-I |
| S100F vs. D97S | -0,5042 | -1,364 to 0,3551 | No | ns | 0,5981 | D-J |
| G149V vs. F229I | -0,1563 | -0,9006 to 0,5879 | No | ns | 0,9991 | E-F |
| G149V vs. Y255X | -0,009828 | -0,7541 to 0,7344 | No | ns | >0,9999 | E-G |
| G149V vs. W383C | -0,0002984 | -0,7445 to 0,7439 | No | ns | >0,9999 | E-H |
| G149V vs. R399H | 0,000713 | -0,7435 to 0,7450 | No | ns | >0,9999 | E-I |
| G149V vs. D97S | -1,725 | -2,529 to -0,9209 | Yes | **** | <0,0001 | E-J |
| F229I vs. Y255X | 0,1465 | -0,5977 to 0,8907 | No | ns | 0,9995 | F-G |
| F229I vs. W383C | 0,156 | -0,5882 to 0,9003 | No | ns | 0,9991 | F-H |
| F229I vs. R399H | 0,1571 | -0,5872 to 0,9013 | No | ns | 0,9991 | F-I |
| F229I vs. D97S | -1,568 | -2,372 to -0,7646 | Yes | **** | <0,0001 | F-J |
| Y255X vs. W383C | 0,009529 | -0,7347 to 0,7538 | No | ns | >0,9999 | G-H |
| Y255X vs. R399H | 0,01054 | -0,7337 to 0,7548 | No | ns | >0,9999 | G-I |
| Y255X vs. D97S | -1,715 | -2,519 to -0,9111 | Yes | **** | <0,0001 | G-J |
| W383C vs. R399H | 0,001011 | -0,7432 to 0,7452 | No | ns | >0,9999 | H-I |
| W383C vs. D97S | -1,725 | -2,528 to -0,9206 | Yes | **** | <0,0001 | H-J |
| R399H vs. D97S | -1,726 | -2,529 to -0,9216 | Yes | **** | <0,0001 | I-J |

**Fig. 1E. NCL CTSD Activity (norm. to CTSD wt), SH-S5Y5 CTSD KO cells**

| | |
|---|---|
| Number of families | 1 |
| Number of comparisons per family | 45 |
| Alpha | 0,05 |

| Tukey's multiple comparisons test | Mean Diff, | 95,00% CI of diff, | Significant? | Summary | Adjusted P Value | |
|---|---|---|---|---|---|---|
| mock vs. wt | -0,9819 | -1,351 to -0,6128 | Yes | **** | <0,0001 | A-B |
| mock vs. A58V | -0,778 | -1,147 to -0,4089 | Yes | **** | <0,0001 | A-C |
| mock vs. S100F | 0,00504 | -0,3936 to 0,4037 | No | ns | >0,9999 | A-D |
| mock vs. G149V | 0,007849 | -0,3612 to 0,3769 | No | ns | >0,9999 | A-E |
| mock vs. F229I | -0,3254 | -0,6944 to 0,04369 | No | ns | 0,1201 | A-F |
| mock vs. Y255X | -0,09271 | -0,4618 to 0,2764 | No | ns | 0,9966 | A-G |
| mock vs. W383C | -0,004512 | -0,3736 to 0,3646 | No | ns | >0,9999 | A-H |
| mock vs. R399H | -0,0289 | -0,3980 to 0,3402 | No | ns | >0,9999 | A-I |
| mock vs. D97S | 0,02077 | -0,3483 to 0,3898 | No | ns | >0,9999 | A-J |
| wt vs. A58V | 0,2039 | -0,1651 to 0,5730 | No | ns | 0,6754 | B-C |

| | | | | | | |
|---|---|---|---|---|---|---|
| wt vs. S100F | 0,9869 | 0,5883 to 1,386 | Yes | **** | <0,0001 | B-D |
| wt vs. G149V | 0,9898 | 0,6207 to 1,359 | Yes | **** | <0,0001 | B-E |
| wt vs. F229I | 0,6565 | 0,2875 to 1,026 | Yes | **** | <0,0001 | B-F |
| wt vs. Y255X | 0,8892 | 0,5201 to 1,258 | Yes | **** | <0,0001 | B-G |
| wt vs. W383C | 0,9774 | 0,6083 to 1,346 | Yes | **** | <0,0001 | B-H |
| wt vs. R399H | 0,953 | 0,5839 to 1,322 | Yes | **** | <0,0001 | B-I |
| wt vs. D97S | 1,003 | 0,6336 to 1,372 | Yes | **** | <0,0001 | B-J |
| A58V vs. S100F | 0,783 | 0,3844 to 1,182 | Yes | **** | <0,0001 | C-D |
| A58V vs. G149V | 0,7858 | 0,4168 to 1,155 | Yes | **** | <0,0001 | C-E |
| A58V vs. F229I | 0,4526 | 0,08352 to 0,8217 | Yes | ** | 0,0076 | C-F |
| A58V vs. Y255X | 0,6853 | 0,3162 to 1,054 | Yes | **** | <0,0001 | C-G |
| A58V vs. W383C | 0,7735 | 0,4044 to 1,143 | Yes | **** | <0,0001 | C-H |
| A58V vs. R399H | 0,7491 | 0,3800 to 1,118 | Yes | **** | <0,0001 | C-I |
| A58V vs. D97S | 0,7987 | 0,4297 to 1,168 | Yes | **** | <0,0001 | C-J |
| S100F vs. G149V | 0,002809 | -0,3958 to 0,4014 | No | ns | >0,9999 | D-E |
| S100F vs. F229I | -0,3304 | -0,7291 to 0,06822 | No | ns | 0,1715 | D-F |
| S100F vs. Y255X | -0,09776 | -0,4964 to 0,3009 | No | ns | 0,9972 | D-G |
| S100F vs. W383C | -0,009552 | -0,4082 to 0,3891 | No | ns | >0,9999 | D-H |
| S100F vs. R399H | -0,03394 | -0,4326 to 0,3647 | No | ns | >0,9999 | D-I |
| S100F vs. D97S | 0,01573 | -0,3829 to 0,4144 | No | ns | >0,9999 | D-J |
| G149V vs. F229I | -0,3332 | -0,7023 to 0,03584 | No | ns | 0,1034 | E-F |
| G149V vs. Y255X | -0,1006 | -0,4696 to 0,2685 | No | ns | 0,9939 | E-G |
| G149V vs. W383C | -0,01236 | -0,3814 to 0,3567 | No | ns | >0,9999 | E-H |
| G149V vs. R399H | -0,03675 | -0,4058 to 0,3323 | No | ns | >0,9999 | E-I |
| G149V vs. D97S | 0,01292 | -0,3561 to 0,3820 | No | ns | >0,9999 | E-J |
| F229I vs. Y255X | 0,2327 | -0,1364 to 0,6017 | No | ns | 0,5063 | F-G |
| F229I vs. W383C | 0,3209 | -0,04820 to 0,6899 | No | ns | 0,1307 | F-H |
| F229I vs. R399H | 0,2965 | -0,07259 to 0,6655 | No | ns | 0,202 | F-I |
| F229I vs. D97S | 0,3462 | -0,02291 to 0,7152 | No | ns | 0,0801 | F-J |
| Y255X vs. W383C | 0,0882 | -0,2809 to 0,4573 | No | ns | 0,9977 | G-H |
| Y255X vs. R399H | 0,06381 | -0,3053 to 0,4329 | No | ns | 0,9998 | G-I |
| Y255X vs. D97S | 0,1135 | -0,2556 to 0,4826 | No | ns | 0,9858 | G-J |
| W383C vs. R399H | -0,02439 | -0,3935 to 0,3447 | No | ns | >0,9999 | H-I |
| W383C vs. D97S | 0,02529 | -0,3438 to 0,3944 | No | ns | >0,9999 | H-J |
| R399H vs. D97S | 0,04967 | -0,3194 to 0,4187 | No | ns | >0,9999 | I-J |

**Fig. 2D. PD Mature Cathepsin D/ Loading Control (norm. to CTSD wt), SH-S5Y5 CTSD KO cells**

| | |
|---|---|
| Number of families | 1 |
| Number of comparisons per family | 21 |
| Alpha | 0,05 |

| Tukey's multiple comparisons test | Mean Diff, | 95,00% CI of diff, | Significant? | Summary | Adjusted P Value | |
|---|---|---|---|---|---|---|
| mock vs. wt | -0,9978 | -2,623 to 0,6272 | No | ns | 0,4455 | A-B |
| mock vs. V95I | -0,9943 | -2,619 to 0,6306 | No | ns | 0,4494 | A-C |
| mock vs. G145V | -1,234 | -2,859 to 0,3908 | No | ns | 0,2203 | A-D |
| mock vs. A239V | -0,4386 | -2,064 to 1,186 | No | ns | 0,9722 | A-E |
| mock vs. R266H | -1,212 | -2,837 to 0,4127 | No | ns | 0,2369 | A-F |
| mock vs. D97S | -1,524 | -3,149 to 0,1008 | No | ns | 0,0754 | A-G |
| wt vs. V95I | 0,003464 | -1,621 to 1,628 | No | ns | >0,9999 | B-C |
| wt vs. G145V | -0,2363 | -1,861 to 1,389 | No | ns | 0,999 | B-D |
| wt vs. A239V | 0,5592 | -1,066 to 2,184 | No | ns | 0,9152 | B-E |
| wt vs. R266H | -0,2145 | -1,839 to 1,410 | No | ns | 0,9994 | B-F |
| wt vs. D97S | -0,5263 | -2,151 to 1,099 | No | ns | 0,9349 | B-G |
| V95I vs. G145V | -0,2398 | -1,865 to 1,385 | No | ns | 0,9989 | C-D |
| V95I vs. A239V | 0,5557 | -1,069 to 2,181 | No | ns | 0,9174 | C-E |
| V95I vs. R266H | -0,218 | -1,843 to 1,407 | No | ns | 0,9993 | C-F |
| V95I vs. D97S | -0,5298 | -2,155 to 1,095 | No | ns | 0,933 | C-G |
| G145V vs. A239V | 0,7955 | -0,8294 to 2,420 | No | ns | 0,6888 | D-E |
| G145V vs. R266H | 0,02184 | -1,603 to 1,647 | No | ns | >0,9999 | D-F |
| G145V vs. D97S | -0,29 | -1,915 to 1,335 | No | ns | 0,9968 | D-G |
| A239V vs. R266H | -0,7737 | -2,399 to 0,8513 | No | ns | 0,7145 | E-F |
| A239V vs. D97S | -1,086 | -2,710 to 0,5394 | No | ns | 0,3506 | E-G |
| R266H vs. D97S | -0,3118 | -1,937 to 1,313 | No | ns | 0,9952 | F-G |

**Fig. 2E. PD CTSD Activity/ Expression Level (norm. to CTSD wt), SH-S5Y5 CTSD KO cells**

| | |
|---|---|
| Number of families | 1 |

| | |
|---|---|
| Number of comparisons per family | 15 |
| Alpha | 0,05 |

| Tukey's multiple comparisons test | Mean Diff, | 95,00% CI of diff, | Significant? | Summary | Adjusted P Value | |
|---|---|---|---|---|---|---|
| wt vs. V95I | -0,008479 | -0,9233 to 0,9064 | No | ns | >0,9999 | A-B |
| wt vs. G145V | 0,2387 | -0,6762 to 1,154 | No | ns | 0,9683 | A-C |
| wt vs. A239V | -1,476 | -2,391 to -0,5616 | Yes | *** | 0,0003 | A-D |
| wt vs. R266H | 0,4011 | -0,5137 to 1,316 | No | ns | 0,7727 | A-E |
| wt vs. D97S | 0,9844 | 0,06960 to 1,899 | Yes | * | 0,0287 | A-F |
| V95I vs. G145V | 0,2472 | -0,6677 to 1,162 | No | ns | 0,9633 | B-C |
| V95I vs. A239V | -1,468 | -2,383 to -0,5531 | Yes | *** | 0,0003 | B-D |
| V95I vs. R266H | 0,4096 | -0,5052 to 1,324 | No | ns | 0,7572 | B-E |
| V95I vs. D97S | 0,9929 | 0,07807 to 1,908 | Yes | * | 0,0268 | B-F |
| G145V vs. A239V | -1,715 | -2,630 to -0,8003 | Yes | **** | <0,0001 | C-D |
| G145V vs. R266H | 0,1624 | -0,7524 to 1,077 | No | ns | 0,9944 | C-E |
| G145V vs. D97S | 0,7457 | -0,1691 to 1,661 | No | ns | 0,1658 | C-F |
| A239V vs. R266H | 1,878 | 0,9627 to 2,792 | Yes | **** | <0,0001 | D-E |
| A239V vs. D97S | 2,461 | 1,546 to 3,376 | Yes | **** | <0,0001 | D-F |
| R266H vs. D97S | 0,5833 | -0,3315 to 1,498 | No | ns | 0,408 | E-F |

**Fig. 3B. PD Mature Cathepsin D/ Loading Control (norm. to CTSD wt), H4 CTSD KO cells**

| | |
|---|---|
| Number of families | 1 |
| Number of comparisons per family | 21 |
| Alpha | 0,05 |

| Tukey's multiple comparisons test | Mean Diff, | 95,00% CI of diff, | Significant? | Summary | Adjusted P Value | |
|---|---|---|---|---|---|---|
| mock vs. wt | -0,8906 | -1,460 to -0,3207 | Yes | *** | 0,0008 | A-B |
| mock vs. V95I | -0,8619 | -1,432 to -0,2920 | Yes | ** | 0,0012 | A-C |
| mock vs. G145V | -0,6532 | -1,223 to -0,08336 | Yes | * | 0,018 | A-D |
| mock vs. A239V | -0,2351 | -0,8049 to 0,3347 | No | ns | 0,8252 | A-E |
| mock vs. R266H | -0,6207 | -1,191 to -0,05085 | Yes | * | 0,027 | A-F |
| mock vs. D97S | -1,007 | -1,577 to -0,4372 | Yes | *** | 0,0002 | A-G |
| wt vs. V95I | 0,02869 | -0,5411 to 0,5985 | No | ns | >0,9999 | B-C |
| wt vs. G145V | 0,2373 | -0,3325 to 0,8072 | No | ns | 0,8189 | B-D |
| wt vs. A239V | 0,6555 | 0,08562 to 1,225 | Yes | * | 0,0175 | B-E |
| wt vs. R266H | 0,2699 | -0,3000 to 0,8397 | No | ns | 0,7193 | B-F |
| wt vs. D97S | -0,1165 | -0,6863 to 0,4534 | No | ns | 0,9933 | B-G |
| V95I vs. G145V | 0,2087 | -0,3612 to 0,7785 | No | ns | 0,8901 | C-D |
| V95I vs. A239V | 0,6268 | 0,05693 to 1,197 | Yes | * | 0,0251 | C-E |
| V95I vs. R266H | 0,2412 | -0,3287 to 0,8110 | No | ns | 0,8081 | C-F |
| V95I vs. D97S | -0,1452 | -0,7150 to 0,4247 | No | ns | 0,9791 | C-G |
| G145V vs. A239V | 0,4181 | -0,1517 to 0,9880 | No | ns | 0,2529 | D-E |
| G145V vs. R266H | 0,03251 | -0,5373 to 0,6024 | No | ns | >0,9999 | D-F |
| G145V vs. D97S | -0,3538 | -0,9237 to 0,2160 | No | ns | 0,4328 | D-G |
| A239V vs. R266H | -0,3856 | -0,9554 to 0,1842 | No | ns | 0,3366 | E-F |
| A239V vs. D97S | -0,7719 | -1,342 to -0,2021 | Yes | ** | 0,0039 | E-G |
| R266H vs. D97S | -0,3863 | -0,9562 to 0,1835 | No | ns | 0,3345 | F-G |

**Fig. 3C. PD CTSD Activity/ Expression Level (norm. to CTSD wt), H4 CTSD KO cells**

| | |
|---|---|
| Number of families | 1 |
| Number of comparisons per family | 15 |
| Alpha | 0,05 |

| Tukey's multiple comparisons test | Mean Diff, | 95,00% CI of diff, | Significant? | Summary | Adjusted P Value | |
|---|---|---|---|---|---|---|
| wt vs. V95I | 0,1824 | -0,09142 to 0,4562 | No | ns | 0,3223 | A-B |
| wt vs. G145V | 0,3987 | 0,1249 to 0,6726 | Yes | ** | 0,0024 | A-C |
| wt vs. A239V | -0,9775 | -1,251 to -0,7037 | Yes | **** | <0,0001 | A-D |
| wt vs. R266H | 0,3916 | 0,1178 to 0,6654 | Yes | ** | 0,0029 | A-E |
| wt vs. D97S | 0,8943 | 0,6205 to 1,168 | Yes | **** | <0,0001 | A-F |
| V95I vs. G145V | 0,2164 | -0,05746 to 0,4902 | No | ns | 0,1725 | B-C |
| V95I vs. A239V | -1,16 | -1,434 to -0,8861 | Yes | **** | <0,0001 | B-D |
| V95I vs. R266H | 0,2092 | -0,06458 to 0,4830 | No | ns | 0,1983 | B-E |
| V95I vs. D97S | 0,7119 | 0,4381 to 0,9857 | Yes | **** | <0,0001 | B-F |
| G145V vs. A239V | -1,376 | -1,650 to -1,102 | Yes | **** | <0,0001 | C-D |
| G145V vs. R266H | -0,007125 | -0,2809 to 0,2667 | No | ns | >0,9999 | C-E |
| G145V vs. D97S | 0,4956 | 0,2218 to 0,7694 | Yes | *** | 0,0002 | C-F |

| | | | | | | |
|---|---|---|---|---|---|---|
| A239V vs. R266H | 1,369 | 1,095 to 1,643 | Yes | **** | <0,0001 | D-E |
| A239V vs. D97S | 1,872 | 1,598 to 2,146 | Yes | **** | <0,0001 | D-F |
| R266H vs. D97S | 0,5027 | 0,2289 to 0,7765 | Yes | *** | 0,0002 | E-F |

**Fig. 3D. PD CTSB Activity (norm to. CTSD wt), H4 CTSD KO cells**

| | |
|---|---|
| Number of families | 1 |
| Number of comparisons per family | 15 |
| Alpha | 0,05 |

| Tukey's multiple comparisons test | Mean Diff, | 95,00% CI of diff, | Significant? | Summary | Adjusted P Value | |
|---|---|---|---|---|---|---|
| wt vs. V95I | 0,155 | -0,1565 to 0,4666 | No | ns | 0,5726 | A-B |
| wt vs. G145V | 0,08848 | -0,2231 to 0,4000 | No | ns | 0,924 | A-C |
| wt vs. A239V | 0,1543 | -0,1572 to 0,4659 | No | ns | 0,5767 | A-D |
| wt vs. R266H | 0,2251 | -0,08642 to 0,5367 | No | ns | 0,221 | A-E |
| wt vs. D97S | 0,3256 | 0,01404 to 0,6372 | Yes | * | 0,0388 | A-F |
| V95I vs. G145V | -0,06654 | -0,3781 to 0,2450 | No | ns | 0,976 | B-C |
| V95I vs. A239V | -0,000674 | -0,3122 to 0,3109 | No | ns | >0,9999 | B-D |
| V95I vs. R266H | 0,07012 | -0,2414 to 0,3817 | No | ns | 0,97 | B-E |
| V95I vs. D97S | 0,1706 | -0,1410 to 0,4821 | No | ns | 0,4788 | B-F |
| G145V vs. A239V | 0,06586 | -0,2457 to 0,3774 | No | ns | 0,977 | C-D |
| G145V vs. R266H | 0,1367 | -0,1749 to 0,4482 | No | ns | 0,6859 | C-E |
| G145V vs. D97S | 0,2371 | -0,07444 to 0,5487 | No | ns | 0,1823 | C-F |
| A239V vs. R266H | 0,0708 | -0,2408 to 0,3824 | No | ns | 0,9688 | D-E |
| A239V vs. D97S | 0,1713 | -0,1403 to 0,4828 | No | ns | 0,4749 | D-F |
| R266H vs. D97S | 0,1005 | -0,2111 to 0,4120 | No | ns | 0,879 | E-F |

**Fig. 3E. PD Gcase Activity (norm to. CTSD wt), H4 CTSD KO cells**

| | |
|---|---|
| Number of families | 1 |
| Number of comparisons per family | 15 |
| Alpha | 0,05 |

| Tukey's multiple comparisons test | Mean Diff, | 95,00% CI of diff, | Significant? | Summary | Adjusted P Value | |
|---|---|---|---|---|---|---|
| wt vs. V95I | -0,1742 | -0,7916 to 0,4431 | No | ns | 0,9257 | A-B |
| wt vs. G145V | -0,05684 | -0,6742 to 0,5605 | No | ns | 0,9995 | A-C |
| wt vs. A239V | -0,1606 | -0,7779 to 0,4568 | No | ns | 0,9459 | A-D |
| wt vs. R266H | -0,05835 | -0,6757 to 0,5590 | No | ns | 0,9994 | A-E |
| wt vs. D97S | -0,01041 | -0,6277 to 0,6069 | No | ns | >0,9999 | A-F |
| V95I vs. G145V | 0,1174 | -0,4999 to 0,7347 | No | ns | 0,9855 | B-C |
| V95I vs. A239V | 0,01368 | -0,6037 to 0,6310 | No | ns | >0,9999 | B-D |
| V95I vs. R266H | 0,1159 | -0,5015 to 0,7332 | No | ns | 0,9863 | B-E |
| V95I vs. D97S | 0,1638 | -0,4535 to 0,7812 | No | ns | 0,9415 | B-F |
| G145V vs. A239V | -0,1037 | -0,7210 to 0,5136 | No | ns | 0,9917 | C-D |
| G145V vs. R266H | -0,001508 | -0,6188 to 0,6158 | No | ns | >0,9999 | C-E |
| G145V vs. D97S | 0,04643 | -0,5709 to 0,6638 | No | ns | 0,9998 | C-F |
| A239V vs. R266H | 0,1022 | -0,5151 to 0,7195 | No | ns | 0,9922 | D-E |
| A239V vs. D97S | 0,1501 | -0,4672 to 0,7675 | No | ns | 0,9587 | D-F |
| R266H vs. D97S | 0,04794 | -0,5694 to 0,6653 | No | ns | 0,9998 | E-F |

**Fig. 3F. Dextran Blue/Celltag700 (Fold Change)**

| | |
|---|---|
| Number of families | 1 |
| Number of comparisons per family | 15 |
| Alpha | 0,05 |

| Tukey's multiple comparisons test | Mean Diff, | 95,00% CI of diff, | Significant? | Summary | Adjusted P Value | |
|---|---|---|---|---|---|---|
| wt vs. V95I | 0,1101 | -0,2714 to 0,4917 | No | ns | 0,9455 | A-B |
| wt vs. R266H | 0,21 | -0,1715 to 0,5916 | No | ns | 0,5466 | A-C |
| wt vs. G145V | -0,05714 | -0,4387 to 0,3244 | No | ns | 0,9971 | A-D |
| wt vs. A234V | -0,1767 | -0,5582 to 0,2049 | No | ns | 0,7111 | A-E |
| wt vs. D97S | -0,1867 | -0,5683 to 0,1949 | No | ns | 0,6626 | A-F |
| V95I vs. R266H | 0,0999 | -0,2986 to 0,4984 | No | ns | 0,9697 | B-C |
| V95I vs. G145V | -0,1673 | -0,5658 to 0,2313 | No | ns | 0,7857 | B-D |
| V95I vs. A234V | -0,2868 | -0,6853 to 0,1117 | No | ns | 0,2651 | B-E |
| V95I vs. D97S | -0,2968 | -0,6954 to 0,1017 | No | ns | 0,2331 | B-F |
| R266H vs. G145V | -0,2672 | -0,6657 to 0,1314 | No | ns | 0,3361 | C-D |
| R266H vs. A234V | -0,3867 | -0,7852 to 0,01185 | No | ns | 0,0609 | C-E |
| R266H vs. D97S | -0,3967 | -0,7953 to 0,001804 | No | ns | 0,0515 | C-F |

| | | | | | | |
|---|---|---|---|---|---|---|
| G145V vs. A234V | -0,1195 | -0,5180 to 0,2790 | No | ns | 0,9365 | D-E |
| G145V vs. D97S | -0,1296 | -0,5281 to 0,2690 | No | ns | 0,913 | D-F |
| A234V vs. D97S | -0,01004 | -0,4086 to 0,3885 | No | ns | >0,9999 | E-F |

**Fig. 4B. PD α-Synuclein Homeostasis/ Loading Control (norm. to CTSD wt). SH-SY5Y CTSD KO cells**

| | |
|---|---|
| Number of families | 1 |
| Number of comparisons per family | 5 |
| Alpha | 0,05 |

| Dunnett's multiple comparisons test | Mean Diff, | 95% CI of diff, | Significant? | Summary | A-? |
|---|---|---|---|---|---|
| wt vs. V95I | -0,043 | -0,3302 to 0,2442 | No | ns | B |
| wt vs. G145V | -0,1163 | -0,4250 to 0,1925 | No | ns | C |
| wt vs. A239V | 0,3053 | 0,008627 to 0,6019 | Yes | * | D |
| wt vs. R266H | -0,05248 | -0,3491 to 0,2442 | No | ns | E |
| wt vs. D97S | -0,1487 | -0,4965 to 0,1992 | No | ns | F |

**Fig. S1C. CTSD Signal Intensity (arbitrary numbers) SH-SY5Y cells**

| Tukey's multiple comparisons test | Mean Diff, | 95,00% CI of diff, | Significant? | Summary | Adjusted P Value | |
|---|---|---|---|---|---|---|
| SH-SY5Y KO vs. SH-SY5Y wt | -0,7686 | -0,8816 to -0,6555 | Yes | **** | <0,0001 | A-B |
| SH-SY5Y KO vs. SH-SY5Y KO + CTSD wt | -0,4754 | -0,5719 to -0,3789 | Yes | **** | <0,0001 | A-C |
| SH-SY5Y wt vs. SH-SY5Y KO + CTSD wt | 0,2932 | 0,1856 to 0,4008 | Yes | **** | <0,0001 | B-C |

**Fig. S3A. NCL Pearson´s Correlation Coeffizient, SH-S5Y5 CTSD KO cells**

| | |
|---|---|
| Number of families | 1 |
| Number of comparisons per family | 36 |
| Alpha | 0,05 |

| Tukey's multiple comparisons test | Mean Diff, | 95,00% CI of diff, | Significant? | Summary | Adjusted P Value | |
|---|---|---|---|---|---|---|
| wt vs. A58V | 0,1082 | -0,05227 to 0,2688 | No | ns | 0,4561 | A-B |
| wt vs. S100F | 0,01288 | -0,1539 to 0,1797 | No | ns | >0,9999 | A-C |
| wt vs. G149V | 0,5162 | 0,3494 to 0,6830 | Yes | **** | <0,0001 | A-D |
| wt vs. F229I | 0,1667 | 0,008805 to 0,3246 | Yes | * | 0,0302 | A-E |
| wt vs. Y255X | 0,5461 | 0,3856 to 0,7066 | Yes | **** | <0,0001 | A-F |
| wt vs. W383C | 0,5504 | 0,3899 to 0,7109 | Yes | **** | <0,0001 | A-G |
| wt vs. R399H | 0,544 | 0,3834 to 0,7045 | Yes | **** | <0,0001 | A-H |
| wt vs. D97S | 0,003566 | -0,1672 to 0,1743 | No | ns | >0,9999 | A-I |
| A58V vs. S100F | -0,09536 | -0,2593 to 0,06858 | No | ns | 0,6552 | B-C |
| A58V vs. G149V | 0,408 | 0,2440 to 0,5719 | Yes | **** | <0,0001 | B-D |
| A58V vs. F229I | 0,05848 | -0,09639 to 0,2133 | No | ns | 0,956 | B-E |
| A58V vs. Y255X | 0,4379 | 0,2803 to 0,5954 | Yes | **** | <0,0001 | B-F |
| A58V vs. W383C | 0,4421 | 0,2846 to 0,5997 | Yes | **** | <0,0001 | B-G |
| A58V vs. R399H | 0,4357 | 0,2782 to 0,5932 | Yes | **** | <0,0001 | B-H |
| A58V vs. D97S | -0,1047 | -0,2726 to 0,06323 | No | ns | 0,5654 | B-I |
| S100F vs. G149V | 0,5033 | 0,3332 to 0,6735 | Yes | **** | <0,0001 | C-D |
| S100F vs. F229I | 0,1538 | -0,007566 to 0,3152 | No | ns | 0,0745 | C-E |
| S100F vs. Y255X | 0,5332 | 0,3693 to 0,6972 | Yes | **** | <0,0001 | C-F |
| S100F vs. W383C | 0,5375 | 0,3736 to 0,7014 | Yes | **** | <0,0001 | C-G |
| S100F vs. R399H | 0,5311 | 0,3671 to 0,6950 | Yes | **** | <0,0001 | C-H |
| S100F vs. D97S | -0,009318 | -0,1833 to 0,1646 | No | ns | >0,9999 | C-I |
| G149V vs. F229I | -0,3495 | -0,5109 to -0,1881 | Yes | **** | <0,0001 | D-E |
| G149V vs. Y255X | 0,02988 | -0,1341 to 0,1938 | No | ns | 0,9997 | D-F |
| G149V vs. W383C | 0,03417 | -0,1298 to 0,1981 | No | ns | 0,9992 | D-G |
| G149V vs. R399H | 0,02774 | -0,1362 to 0,1917 | No | ns | 0,9998 | D-H |
| G149V vs. D97S | -0,5127 | -0,6866 to -0,3387 | Yes | **** | <0,0001 | D-I |
| F229I vs. Y255X | 0,3794 | 0,2245 to 0,5342 | Yes | **** | <0,0001 | E-F |
| F229I vs. W383C | 0,3837 | 0,2288 to 0,5385 | Yes | **** | <0,0001 | E-G |
| F229I vs. R399H | 0,3772 | 0,2224 to 0,5321 | Yes | **** | <0,0001 | E-H |
| F229I vs. D97S | -0,1632 | -0,3286 to 0,002273 | No | ns | 0,0563 | E-I |
| Y255X vs. W383C | 0,004286 | -0,1532 to 0,1618 | No | ns | >0,9999 | F-G |
| Y255X vs. R399H | -0,002143 | -0,1597 to 0,1554 | No | ns | >0,9999 | F-H |
| Y255X vs. D97S | -0,5425 | -0,7104 to -0,3746 | Yes | **** | <0,0001 | F-I |
| W383C vs. R399H | -0,006429 | -0,1639 to 0,1511 | No | ns | >0,9999 | G-H |
| W383C vs. D97S | -0,5468 | -0,7147 to -0,3789 | Yes | **** | <0,0001 | G-I |

| | | | | | | |
|---|---|---|---|---|---|---|
| R399H vs. D97S | -0,5404 | -0,7083 to -0,3725 | Yes | **** | <0,0001 | H-I |

**Fig. S3B NCL Vesicles per Cell, SH-S5Y5 CTSD KO cells**

| | |
|---|---|
| Number of families | 1 |
| Number of comparisons per family | 45 |
| Alpha | 0,05 |

| Tukey's multiple comparisons test | Mean Diff, | 95,00% CI of diff, | Significant? | Summary | Adjusted P Value | |
|---|---|---|---|---|---|---|
| mock vs. wt | 0,5294 | -3,689 to 4,748 | No | ns | >0,9999 | A-B |
| mock vs. A58V | -3,108 | -7,268 to 1,052 | No | ns | 0,334 | A-C |
| mock vs. S100F | -7,941 | -12,38 to -3,502 | Yes | **** | <0,0001 | A-D |
| mock vs. G149V | -3,254 | -7,538 to 1,031 | No | ns | 0,311 | A-E |
| mock vs. F229I | 0,3529 | -3,866 to 4,572 | No | ns | >0,9999 | A-F |
| mock vs. Y255X | -0,6555 | -5,095 to 3,784 | No | ns | >0,9999 | A-G |
| mock vs. W383C | -0,5728 | -4,679 to 3,534 | No | ns | >0,9999 | A-H |
| mock vs. R399H | -1,066 | -5,350 to 3,218 | No | ns | 0,9985 | A-I |
| mock vs. D97S | -1,235 | -5,454 to 2,983 | No | ns | 0,9949 | A-J |
| wt vs. A58V | -3,637 | -7,797 to 0,5225 | No | ns | 0,1433 | B-C |
| wt vs. S100F | -8,471 | -12,91 to -4,032 | Yes | **** | <0,0001 | B-D |
| wt vs. G149V | -3,783 | -8,067 to 0,5011 | No | ns | 0,1341 | B-E |
| wt vs. F229I | -0,1765 | -4,395 to 4,042 | No | ns | >0,9999 | B-F |
| wt vs. Y255X | -1,185 | -5,624 to 3,254 | No | ns | 0,9974 | B-G |
| wt vs. W383C | -1,102 | -5,208 to 3,004 | No | ns | 0,9973 | B-H |
| wt vs. R399H | -1,596 | -5,880 to 2,689 | No | ns | 0,9721 | B-I |
| wt vs. D97S | -1,765 | -5,983 to 2,454 | No | ns | 0,9419 | B-J |
| A58V vs. S100F | -4,833 | -9,216 to -0,4503 | Yes | * | 0,0184 | C-D |
| A58V vs. G149V | -0,1458 | -4,372 to 4,080 | No | ns | >0,9999 | C-E |
| A58V vs. F229I | 3,461 | -0,6990 to 7,621 | No | ns | 0,195 | C-F |
| A58V vs. Y255X | 2,452 | -1,931 to 6,835 | No | ns | 0,7366 | C-G |
| A58V vs. W383C | 2,535 | -1,511 to 6,581 | No | ns | 0,5922 | C-H |
| A58V vs. R399H | 2,042 | -2,184 to 6,268 | No | ns | 0,869 | C-I |
| A58V vs. D97S | 1,873 | -2,287 to 6,032 | No | ns | 0,9108 | C-J |
| S100F vs. G149V | 4,688 | 0,1862 to 9,189 | Yes | * | 0,0339 | D-E |
| S100F vs. F229I | 8,294 | 3,855 to 12,73 | Yes | **** | <0,0001 | D-F |
| S100F vs. Y255X | 7,286 | 2,637 to 11,93 | Yes | **** | <0,0001 | D-G |
| S100F vs. W383C | 7,368 | 3,036 to 11,70 | Yes | **** | <0,0001 | D-H |
| S100F vs. R399H | 6,875 | 2,374 to 11,38 | Yes | *** | 0,0001 | D-I |
| S100F vs. D97S | 6,706 | 2,267 to 11,14 | Yes | *** | 0,0001 | D-J |
| G149V vs. F229I | 3,607 | -0,6776 to 7,891 | No | ns | 0,1819 | E-F |
| G149V vs. Y255X | 2,598 | -1,903 to 7,099 | No | ns | 0,7002 | E-G |
| G149V vs. W383C | 2,681 | -1,493 to 6,854 | No | ns | 0,5568 | E-H |
| G149V vs. R399H | 2,188 | -2,161 to 6,536 | No | ns | 0,839 | E-I |
| G149V vs. D97S | 2,018 | -2,266 to 6,303 | No | ns | 0,8853 | E-J |
| F229I vs. Y255X | -1,008 | -5,447 to 3,431 | No | ns | 0,9993 | F-G |
| F229I vs. W383C | -0,9257 | -5,032 to 3,181 | No | ns | 0,9993 | F-H |
| F229I vs. R399H | -1,419 | -5,703 to 2,865 | No | ns | 0,9874 | F-I |
| F229I vs. D97S | -1,588 | -5,807 to 2,631 | No | ns | 0,97 | F-J |
| Y255X vs. W383C | 0,08271 | -4,250 to 4,415 | No | ns | >0,9999 | G-H |
| Y255X vs. R399H | -0,4107 | -4,912 to 4,091 | No | ns | >0,9999 | G-I |
| Y255X vs. D97S | -0,5798 | -5,019 to 3,859 | No | ns | >0,9999 | G-J |
| W383C vs. R399H | -0,4934 | -4,667 to 3,680 | No | ns | >0,9999 | H-I |
| W383C vs. D97S | -0,6625 | -4,769 to 3,444 | No | ns | >0,9999 | H-J |
| R399H vs. D97S | -0,1691 | -4,453 to 4,115 | No | ns | >0,9999 | I-J |

**Fig. S3C. NCL Average Vesicle Size per Cell, SH-S5Y5 CTSD KO cells**

| | |
|---|---|
| Number of families | 1 |
| Number of comparisons per family | 45 |
| Alpha | 0,05 |

| Tukey's multiple comparisons test | Mean Diff, | 95,00% CI of diff, | Significant? | Summary | Adjusted P Value | |
|---|---|---|---|---|---|---|
| mock vs. wt | 0,7563 | 0,4430 to 1,070 | Yes | **** | <0,0001 | A-B |
| mock vs. A58V | 0,7662 | 0,4573 to 1,075 | Yes | **** | <0,0001 | A-C |
| mock vs. S100F | 0,7077 | 0,3780 to 1,037 | Yes | **** | <0,0001 | A-D |
| mock vs. G149V | 0,4637 | 0,1455 to 0,7819 | Yes | *** | 0,0003 | A-E |
| mock vs. F229I | 0,2642 | -0,04918 to 0,5775 | No | ns | 0,1804 | A-F |
| mock vs. Y255X | 0,2975 | -0,03224 to 0,6272 | No | ns | 0,1155 | A-G |
| mock vs. W383C | 0,1694 | -0,1356 to 0,4744 | No | ns | 0,7447 | A-H |

| | | | | | | |
|---|---|---|---|---|---|---|
| mock vs. R399H | 0,4202 | 0,1020 to 0,7384 | Yes | ** | 0,0015 | A-I |
| mock vs. D97S | 0,7422 | 0,4288 to 1,056 | Yes | **** | <0,0001 | A-J |
| wt vs. A58V | 0,009877 | -0,2991 to 0,3188 | No | ns | >0,9999 | B-C |
| wt vs. S100F | -0,04862 | -0,3783 to 0,2811 | No | ns | >0,9999 | B-D |
| wt vs. G149V | -0,2926 | -0,6108 to 0,02558 | No | ns | 0,1004 | B-E |
| wt vs. F229I | -0,4922 | -0,8055 to -0,1788 | Yes | **** | <0,0001 | B-F |
| wt vs. Y255X | -0,4589 | -0,7886 to -0,1292 | Yes | *** | 0,0006 | B-G |
| wt vs. W383C | -0,5869 | -0,8919 to -0,2820 | Yes | **** | <0,0001 | B-H |
| wt vs. R399H | -0,3362 | -0,6544 to -0,01795 | Yes | * | 0,0293 | B-I |
| wt vs. D97S | -0,01416 | -0,3275 to 0,2992 | No | ns | >0,9999 | B-J |
| A58V vs. S100F | -0,0585 | -0,3840 to 0,2670 | No | ns | 0,9999 | C-D |
| A58V vs. G149V | -0,3025 | -0,6164 to 0,01139 | No | ns | 0,0692 | C-E |
| A58V vs. F229I | -0,5021 | -0,8110 to -0,1931 | Yes | **** | <0,0001 | C-F |
| A58V vs. Y255X | -0,4688 | -0,7943 to -0,1432 | Yes | *** | 0,0003 | C-G |
| A58V vs. W383C | -0,5968 | -0,8973 to -0,2963 | Yes | **** | <0,0001 | C-H |
| A58V vs. R399H | -0,346 | -0,6599 to -0,03214 | Yes | * | 0,0184 | C-I |
| A58V vs. D97S | -0,02404 | -0,3330 to 0,2849 | No | ns | >0,9999 | C-J |
| S100F vs. G149V | -0,244 | -0,5783 to 0,09032 | No | ns | 0,3678 | D-E |
| S100F vs. F229I | -0,4436 | -0,7733 to -0,1139 | Yes | ** | 0,0011 | D-F |
| S100F vs. Y255X | -0,4103 | -0,7556 to -0,06498 | Yes | ** | 0,0073 | D-G |
| S100F vs. W383C | -0,5383 | -0,8601 to -0,2165 | Yes | **** | <0,0001 | D-H |
| S100F vs. R399H | -0,2875 | -0,6219 to 0,04679 | No | ns | 0,1595 | D-I |
| S100F vs. D97S | 0,03446 | -0,2952 to 0,3642 | No | ns | >0,9999 | D-J |
| G149V vs. F229I | -0,1996 | -0,5178 to 0,1186 | No | ns | 0,591 | E-F |
| G149V vs. Y255X | -0,1663 | -0,5006 to 0,1681 | No | ns | 0,848 | E-G |
| G149V vs. W383C | -0,2943 | -0,6043 to 0,01566 | No | ns | 0,0782 | E-H |
| G149V vs. R399H | -0,04353 | -0,3665 to 0,2795 | No | ns | >0,9999 | E-I |
| G149V vs. D97S | 0,2785 | -0,03974 to 0,5967 | No | ns | 0,1425 | E-J |
| F229I vs. Y255X | 0,03329 | -0,2964 to 0,3630 | No | ns | >0,9999 | F-G |
| F229I vs. W383C | -0,09476 | -0,3997 to 0,2102 | No | ns | 0,992 | F-H |
| F229I vs. R399H | 0,156 | -0,1622 to 0,4742 | No | ns | 0,8585 | F-I |
| F229I vs. D97S | 0,478 | 0,1647 to 0,7914 | Yes | *** | 0,0001 | F-J |
| Y255X vs. W383C | -0,1281 | -0,4498 to 0,1937 | No | ns | 0,9572 | G-H |
| Y255X vs. R399H | 0,1227 | -0,2116 to 0,4571 | No | ns | 0,9746 | G-I |
| Y255X vs. D97S | 0,4447 | 0,1150 to 0,7744 | Yes | ** | 0,0011 | G-J |
| W383C vs. R399H | 0,2508 | -0,05918 to 0,5608 | No | ns | 0,2279 | H-I |
| W383C vs. D97S | 0,5728 | 0,2678 to 0,8778 | Yes | **** | <0,0001 | H-J |
| R399H vs. D97S | 0,322 | 0,003789 to 0,6402 | Yes | * | 0,0448 | I-J |

**Fig. S5A. PD Pearson´s Correlation Coeffizient, SH-S5Y5 CTSD KO cells**

| | |
|---|---|
| Number of families | 1 |
| Number of comparisons per family | 15 |
| Alpha | 0,05 |

| Tukey's multiple comparisons test | Mean Diff, | 95,00% CI of diff, | Significant? | Summary | Adjusted P Value | |
|---|---|---|---|---|---|---|
| wt vs. V95I | 0,05523 | -0,09947 to 0,2099 | No | ns | 0,9001 | A-B |
| wt vs. G145V | -0,0015 | -0,1450 to 0,1420 | No | ns | >0,9999 | A-C |
| wt vs. A239V | -0,02536 | -0,1711 to 0,1204 | No | ns | 0,9956 | A-D |
| wt vs. R266H | -0,01295 | -0,1676 to 0,1417 | No | ns | 0,9999 | A-E |
| wt vs. D97S | -0,04114 | -0,1958 to 0,1136 | No | ns | 0,97 | A-F |
| V95I vs. G145V | -0,05673 | -0,2038 to 0,09038 | No | ns | 0,8668 | B-C |
| V95I vs. A239V | -0,08058 | -0,2299 to 0,06873 | No | ns | 0,6126 | B-D |
| V95I vs. R266H | -0,06818 | -0,2262 to 0,08984 | No | ns | 0,8026 | B-E |
| V95I vs. D97S | -0,09636 | -0,2544 to 0,06166 | No | ns | 0,48 | B-F |
| G145V vs. A239V | -0,02386 | -0,1616 to 0,1139 | No | ns | 0,9957 | C-D |
| G145V vs. R266H | -0,01145 | -0,1586 to 0,1357 | No | ns | >0,9999 | C-E |
| G145V vs. D97S | -0,03964 | -0,1867 to 0,1075 | No | ns | 0,9683 | C-F |
| A239V vs. R266H | 0,0124 | -0,1369 to 0,1617 | No | ns | 0,9999 | D-E |
| A239V vs. D97S | -0,01578 | -0,1651 to 0,1335 | No | ns | 0,9996 | D-F |
| R266H vs. D97S | -0,02818 | -0,1862 to 0,1298 | No | ns | 0,9951 | E-F |

**Fig. S5B. PD Vesicles per Cell, SH-S5Y5 CTSD KO cells**

| | |
|---|---|
| Number of families | 1 |
| Number of comparisons per family | 21 |
| Alpha | 0,05 |

| Tukey's multiple comparisons test | Mean Diff, | 95,00% CI of diff, | Significant? | Summary | Adjusted P Value | |
|---|---|---|---|---|---|---|
| mock vs. wt | -0,1891 | -6,034 to 5,656 | No | ns | >0,9999 | A-B |
| mock vs. V97S | -2,546 | -8,391 to 3,299 | No | ns | 0,8449 | A-C |
| mock vs. G145V | -3,93 | -9,571 to 1,711 | No | ns | 0,3623 | A-D |
| mock vs. A239V | -4,851 | -10,59 to 0,8862 | No | ns | 0,1548 | A-E |
| mock vs. R266H | -4,572 | -10,84 to 1,695 | No | ns | 0,3069 | A-F |
| mock vs. D97S | -1,765 | -7,320 to 3,790 | No | ns | 0,9619 | A-G |
| wt vs. V97S | -2,357 | -8,478 to 3,764 | No | ns | 0,9074 | B-C |
| wt vs. G145V | -3,741 | -9,668 to 2,186 | No | ns | 0,4849 | B-D |
| wt vs. A239V | -4,662 | -10,68 to 1,357 | No | ns | 0,2395 | B-E |
| wt vs. R266H | -4,383 | -10,91 to 2,142 | No | ns | 0,4074 | B-F |
| wt vs. D97S | -1,576 | -7,421 to 4,269 | No | ns | 0,9833 | B-G |
| V97S vs. G145V | -1,384 | -7,311 to 4,543 | No | ns | 0,9921 | C-D |
| V97S vs. A239V | -2,305 | -8,323 to 3,714 | No | ns | 0,9096 | C-E |
| V97S vs. R266H | -2,026 | -8,551 to 4,499 | No | ns | 0,9659 | C-F |
| V97S vs. D97S | 0,7815 | -5,064 to 6,627 | No | ns | 0,9997 | C-G |
| G145V vs. A239V | -0,9208 | -6,741 to 4,900 | No | ns | 0,9991 | D-E |
| G145V vs. R266H | -0,642 | -6,985 to 5,701 | No | ns | >0,9999 | D-F |
| G145V vs. D97S | 2,165 | -3,476 to 7,807 | No | ns | 0,9086 | D-G |
| A239V vs. R266H | 0,2788 | -6,150 to 6,708 | No | ns | >0,9999 | E-F |
| A239V vs. D97S | 3,086 | -2,651 to 8,823 | No | ns | 0,67 | E-G |
| R266H vs. D97S | 2,807 | -3,459 to 9,074 | No | ns | 0,8269 | F-G |

**Fig. S5C. PD Average Vesicle Size per Cell, SH-S5Y5 CTSD KO cells**

| | |
|---|---|
| Number of families | 1 |
| Number of comparisons per family | 21 |
| Alpha | 0,05 |

| Tukey's multiple comparisons test | Mean Diff, | 95,00% CI of diff, | Significant? | Summary | Adjusted P Value | |
|---|---|---|---|---|---|---|
| mock vs. wt | 0,3033 | 0,1336 to 0,4729 | Yes | **** | <0,0001 | A-B |
| mock vs. V97S | 0,2862 | 0,1166 to 0,4558 | Yes | **** | <0,0001 | A-C |
| mock vs. G145V | 0,3271 | 0,1634 to 0,4908 | Yes | **** | <0,0001 | A-D |
| mock vs. A239V | 0,299 | 0,1325 to 0,4655 | Yes | **** | <0,0001 | A-E |
| mock vs. R266H | 0,3743 | 0,1925 to 0,5562 | Yes | **** | <0,0001 | A-F |
| mock vs. D97S | 0,2965 | 0,1353 to 0,4577 | Yes | **** | <0,0001 | A-G |
| wt vs. V97S | -0,01706 | -0,1947 to 0,1606 | No | ns | >0,9999 | B-C |
| wt vs. G145V | 0,02381 | -0,1482 to 0,1958 | No | ns | 0,9996 | B-D |
| wt vs. A239V | -0,004274 | -0,1789 to 0,1704 | No | ns | >0,9999 | B-E |
| wt vs. R266H | 0,07107 | -0,1183 to 0,2604 | No | ns | 0,9173 | B-F |
| wt vs. D97S | -0,006765 | -0,1764 to 0,1629 | No | ns | >0,9999 | B-G |
| V97S vs. G145V | 0,04087 | -0,1311 to 0,2129 | No | ns | 0,9914 | C-D |
| V97S vs. A239V | 0,01279 | -0,1619 to 0,1874 | No | ns | >0,9999 | C-E |
| V97S vs. R266H | 0,08813 | -0,1012 to 0,2775 | No | ns | 0,7998 | C-F |
| V97S vs. D97S | 0,01029 | -0,1593 to 0,1799 | No | ns | >0,9999 | C-G |
| G145V vs. A239V | -0,02809 | -0,1970 to 0,1408 | No | ns | 0,9988 | D-E |
| G145V vs. R266H | 0,04726 | -0,1368 to 0,2313 | No | ns | 0,987 | D-F |
| G145V vs. D97S | -0,03058 | -0,1943 to 0,1331 | No | ns | 0,9977 | D-G |
| A239V vs. R266H | 0,07534 | -0,1112 to 0,2619 | No | ns | 0,8863 | E-F |
| A239V vs. D97S | -0,002492 | -0,1690 to 0,1640 | No | ns | >0,9999 | E-G |
| R266H vs. D97S | -0,07783 | -0,2597 to 0,1040 | No | ns | 0,8555 | F-G |

**Fig. S5D. Mature Cathepsin D/ Immature Cathepsin D (norm. to CTSD wt), SH-S5Y5 CTSD KO cells**

| | |
|---|---|
| Number of families | 1 |
| Number of comparisons per family | 15 |
| Alpha | 0,05 |

| Tukey's multiple comparisons test | Mean Diff, | 95,00% CI of diff, | Significant? | Summary | Adjusted P Value | |
|---|---|---|---|---|---|---|
| wt vs. V95I | 0,3254 | -0,8597 to 1,510 | No | ns | 0,9622 | B-C |
| wt vs. G145V | -0,3004 | -1,485 to 0,8847 | No | ns | 0,9732 | B-D |
| wt vs. A239V | -0,448 | -1,633 to 0,7370 | No | ns | 0,8667 | B-E |
| wt vs. R266H | -0,2634 | -1,449 to 0,9216 | No | ns | 0,985 | B-F |
| wt vs. D97S | -1,093 | -2,278 to 0,09242 | No | ns | 0,0859 | B-G |
| V95I vs. G145V | -0,6258 | -1,811 to 0,5593 | No | ns | 0,6181 | C-D |
| V95I vs. A239V | -0,7734 | -1,959 to 0,4116 | No | ns | 0,3884 | C-E |
| V95I vs. R266H | -0,5888 | -1,774 to 0,5962 | No | ns | 0,6765 | C-F |
| V95I vs. D97S | -1,418 | -2,603 to -0,2330 | Yes | * | 0,0109 | C-G |
| G145V vs. A239V | -0,1477 | -1,333 to 1,037 | No | ns | 0,999 | D-E |

| | | | | | | |
|---|---|---|---|---|---|---|
| G145V vs. R266H | 0,03695 | -1,148 to 1,222 | No | ns | >0,9999 | D-F |
| G145V vs. D97S | -0,7923 | -1,977 to 0,3928 | No | ns | 0,3618 | D-G |
| A239V vs. R266H | 0,1846 | -1,000 to 1,370 | No | ns | 0,9971 | E-F |
| A239V vs. D97S | -0,6446 | -1,830 to 0,5405 | No | ns | 0,588 | E-G |
| R266H vs. D97S | -0,8292 | -2,014 to 0,3559 | No | ns | 0,3126 | F-G |

**Fig. S5E. Mature Cathepsin D/ Immature Cathepsin D (norm. to CTSD wt), H4 CTSD KO cells**

| | |
|---|---|
| Number of families | 1 |
| Number of comparisons per family | 15 |
| Alpha | 0,05 |

| Tukey's multiple comparisons test | Mean Diff, | 95,00% CI of diff, | Significant? | Summary | Adjusted P Value | |
|---|---|---|---|---|---|---|
| wt vs. V95I | -0,4564 | -1,727 to 0,8138 | No | ns | 0,8539 | A-B |
| wt vs. G145V | -0,9487 | -2,219 to 0,3215 | No | ns | 0,2142 | A-C |
| wt vs. A239V | -0,8643 | -2,236 to 0,5076 | No | ns | 0,374 | A-D |
| wt vs. R266H | -0,3723 | -1,643 to 0,8978 | No | ns | 0,9309 | A-E |
| wt vs. D97S | -1,693 | -2,963 to -0,4225 | Yes | ** | 0,0058 | A-F |
| V95I vs. G145V | -0,4923 | -1,763 to 0,7779 | No | ns | 0,8117 | B-C |
| V95I vs. A239V | -0,4079 | -1,780 to 0,9640 | No | ns | 0,927 | B-D |
| V95I vs. R266H | 0,08406 | -1,186 to 1,354 | No | ns | >0,9999 | B-E |
| V95I vs. D97S | -1,236 | -2,506 to 0,03395 | No | ns | 0,059 | B-F |
| G145V vs. A239V | 0,08438 | -1,288 to 1,456 | No | ns | >0,9999 | C-D |
| G145V vs. R266H | 0,5764 | -0,6938 to 1,847 | No | ns | 0,6972 | C-E |
| G145V vs. D97S | -0,7439 | -2,014 to 0,5263 | No | ns | 0,4495 | C-F |
| A239V vs. R266H | 0,492 | -0,8800 to 1,864 | No | ns | 0,8549 | D-E |
| A239V vs. D97S | -0,8283 | -2,200 to 0,5437 | No | ns | 0,4179 | D-F |
| R266H vs. D97S | -1,32 | -2,590 to -0,05011 | Yes | * | 0,0391 | E-F |

**Fig. S6B. a-Synuclein Signal Western Blot (norm. to mock)**

| Dunnett's multiple comparisons test | Mean Diff, | 95,00% CI of diff, | Significant? | Summary | Adjusted P Va | A-? |
|---|---|---|---|---|---|---|
| mock vs. BafA1 | -1,165 | -2,089 to -0,2399 | Yes | * | 0,0216 | B |
| mock vs. PepA | -1,518 | -2,443 to -0,5937 | Yes | ** | 0,0074 | C |
